# Kinetics of Antigen Expression and Epitope Presentation during Virus Infection

**DOI:** 10.1371/journal.ppat.1003129

**Published:** 2013-01-31

**Authors:** Nathan P. Croft, Stewart A. Smith, Yik Chun Wong, Chor Teck Tan, Nadine L. Dudek, Inge E. A. Flesch, Leon C. W. Lin, David C. Tscharke, Anthony W. Purcell

**Affiliations:** 1 Department of Biochemistry and Molecular Biology, Monash University, Clayton, Victoria, Australia; 2 Research School of Biology, The Australian National University, Canberra, Australian Capital Territory, Australia; University of Pennsylvania, United States of America

## Abstract

Current knowledge about the dynamics of antigen presentation to T cells during viral infection is very poor despite being of fundamental importance to our understanding of anti-viral immunity. Here we use an advanced mass spectrometry method to simultaneously quantify the presentation of eight vaccinia virus peptide-MHC complexes (epitopes) on infected cells and the amounts of their source antigens at multiple times after infection. The results show a startling 1000-fold range in abundance as well as strikingly different kinetics across the epitopes monitored. The tight correlation between onset of protein expression and epitope display for most antigens provides the strongest support to date that antigen presentation is largely linked to translation and not later degradation of antigens. Finally, we show a complete disconnect between the epitope abundance and immunodominance hierarchy of these eight epitopes. This study highlights the complexity of viral antigen presentation by the host and demonstrates the weakness of simple models that assume total protein levels are directly linked to epitope presentation and immunogenicity.

## Author Summary

A major mechanism for the detection of virus infection is the recognition by T cells of short peptide fragments (epitopes) derived from the degradation of intracellular proteins presented at the cell surface in a complex with class I MHC. Whilst the mechanics of antigen degradation and the loading of peptides onto MHC are now well understood, the kinetics of epitope presentation have only been studied for individual model antigens. We addressed this issue by studying vaccinia virus, best known as the smallpox vaccine, using advanced mass spectrometry. Precise and simultaneous quantification of multiple peptide-MHC complexes showed that the surface of infected cells provides a surprisingly dynamic landscape from the point of view of anti-viral T cells. Further, concurrent measurement of virus protein levels demonstrated that in most cases, peak presentation of epitopes occurs at the same time or precedes the time of maximum protein build up. Finally, we found a complete disconnect between the abundance of epitopes on infected cells and the size of the responding T cell populations. These data provide new insights into how virus infected cells are seen by T cells, which is crucial to our understanding of anti-viral immunity and development of vaccines.

## Introduction

The presentation of virus peptides (epitopes) to CD8^+^ T cells plays a pivotal role in anti-viral immunity. Recognition of these epitopes presented on MHC class I drives CD8^+^ T cell priming following interactions with professional antigen presenting cells (APC) and subsequently allows control of infection through killing of infected cells and secretion of cytokines. The process of MHC class I antigen presentation is complex and multi-staged. It starts with degradation of polypeptides, typically by the proteasome, followed by transport to the ER, loading onto MHC class I and finally egress to the cell surface [1]. Along the way other proteases and chaperones refine the peptides and perform quality control functions on peptide-MHC complexes (pMHC) [2]. Surprisingly, despite the large coding capacity and therefore antigenic potential of many viruses, CD8^+^ T cell responses are often skewed towards a small number of peptides in a phenomenon known as immunodominance [3]. This is exemplified by studies of humans and animals infected with large, complex dsDNA viruses, such as herpes- and poxviruses, where reproducible CD8^+^ immunodominance hierarchies emerge. For example, up to 20% of the CD8^+^ T cell response following infection of C57BL/6 mice with vaccinia virus (VACV) is directed towards a single immunodominant epitope and a handful of subdominant specificities account for much of the remainder [4], [5]. Further, while MHC class I antigen presentation is well understood in principle [6] and bioinformatic predictions of MHC class I binding are often highly refined [7], prediction of antigenicity and immunogenicity have remained elusive.

In part this gap remains because kinetic studies to date have focused on single peptides [8] and broader scale studies of antigenicity have been limited to single time points [9]–[11]. This has reflected limitations of technology in that the best reagents for quantifying antigen presentation have been the few monoclonal antibodies generated to date that recognise specific pMHC complexes [8], [12]–[15]. Proteome-wide biochemical approaches have typically required prohibitively large numbers of cells (1×10^9^ and greater) restricting experiments to single time points [16], [17] . Although we have good examples showing the diversity of native virus epitopes presented and we know the consequences of manipulating expression levels and even translation rates for presentation of model antigens [8], [18], this information remains disconnected. As a consequence, while it is clear that increasing expression of a given antigen leads to higher presentation of epitopes, it is not known whether antigen expression level *per se* is a useful predictor of likely antigenicity across different viral proteins. Further, whether bulk protein abundance or expression levels correlate best with production of epitopes as a general rule is not known. Indeed, several recent studies have highlighted the diversity of source for MHC class I bound peptides and have implicated both products of translational infidelity (defective ribosome initiation products (DRiPs)) [10], [19]–[22] as well as mature proteins [23]. For instance, some biochemical surveys of epitope versus transcript or steady-state antigen abundance suggest these are closely related at single time points [16], [24]. However, most epitopes studied in detail are shown to be the products of recent translation and therefore need not be related to final antigen abundance [25]–[28]. Only studies that can link the kinetics of antigen synthesis and accumulation with epitope presentation for multiple native virus proteins will allow general conclusions to be drawn. Finally, antigen expression levels can be linked to immunogenicity for model antigens, but again whether this is useful for evaluating whole viral proteomes has not been approached.

Here we present the first study that links the kinetics of virus protein build up and CD8^+^ T cell epitope presentation for multiple pMHC complexes. We used vaccinia virus, best known as the vaccine used to eradicate smallpox, taking advantage of its robust *in vitro* infections and a well characterised CD8^+^ T cell epitope hierarchy [4], [5]. In addition there is good evidence that anti-VACV CD8^+^ T cells are directly primed by infected APC making this an ideal choice to study antigen presentation *in vitro* [29]–[31]. The abundance of 8 VACV epitopes was quantified simultaneously at multiple times after infection using the multiple reaction monitoring approach to tandem mass spectrometry [32]. The same method was applied in parallel to determine relative abundance of the relevant virus proteins using filter assisted sample preparation and whole cell tryptic digestion [33]. Together, these data provide an unparalleled insight into the dynamic nature of antigen presentation on class I during a virus replication cycle. Further they provide the most compelling evidence to date of the direct correlation between the timing of virus antigen expression and the appearance of epitopes derived from the same protein. Finally, while we can now add kinetics to our description of epitope presentation for multiple epitopes, these biochemical data still fail to predict the hierarchy of immunodominance in responding CD8^+^ T cell responses.

## Results

### Antigen presentation during viral infection is complex and cannot be described by a single epitope

**Figure 1:**
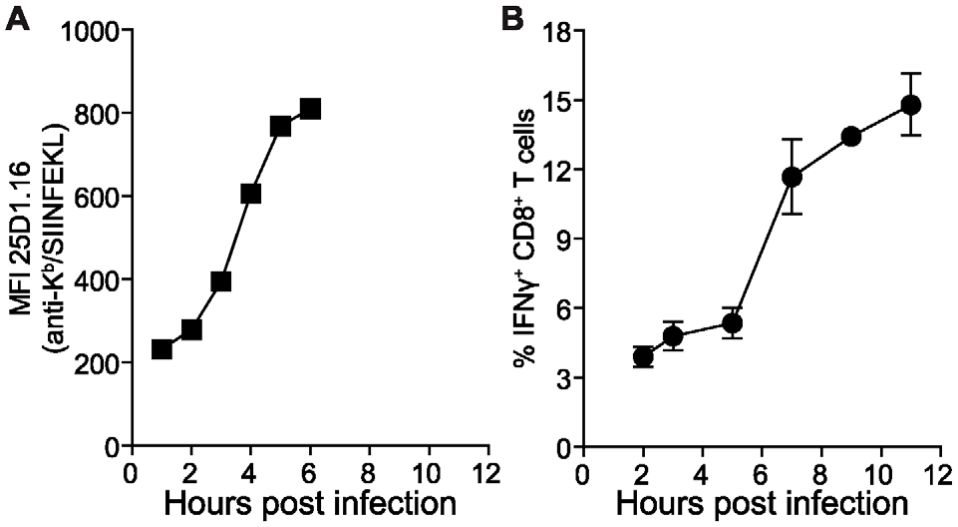
Antigen presentation during virus infection is complex and cannot be described by a single epitope. DC2.4 cells were infected with VACV strain WR-NP-S-GFP (A) or WR (B) and presentation of MHC class I peptides detected at various times. A) K^b^-SIINFEKL presentation levels determined by staining with mAb 25-D1.16 and flow cytometry. Data are representative multiple experiments. B) Presentation of bulk native VACV epitopes determined by incubation with VACV-immune splenocytes and detecting activation by staining for surface CD8^+^ and intracellular IFNγ. Means and SEM of triplicates are shown and the data are representative of two independent experiments.

Previous studies aimed at understanding antigen presentation kinetics have focussed on single epitopes, most commonly the model peptide SIINFEKL (presented by H-2K^b^) expressed from recombinant viruses, including VACV. Whilst these experiments have yielded much useful mechanistic insight, it is not clear whether kinetic data generated are representative of virus epitopes in general. To examine this issue, we first recapitulated published data showing the rapid rise of H-2K^b^-SIINFEKL complexes on cells infected with a recombinant VACV strain WR-NP-S-GFP [8], [13]. This virus expresses a chimera in which SIINFEKL is sandwiched between influenza virus nucleoprotein and enhanced green fluorescent protein [8], [34]. DC2.4 cells, a dendritic cell-like line derived from C57BL/6 mice, were infected at a multiplicity of 10 pfu per cell and presentation of K^b^-SIINFEKL complexes measured using the mAb 25D1.16 and flow cytometry at various times (Figure 1A). Consistent with previous work that typically used L-K^b^ cells, in DC2.4 K^b^-SIINFEKL complexes rose rapidly after infection and began to plateau by 6 hours post infection (hpi). To test if the kinetics observed for K^b^-SIINFEKL complexes is representative of all VACV epitopes we used polyclonal T cells isolated from infected mice since monoclonal antibodies to VACV epitope-MHC complexes are not available. If all VACV antigen presentation is like K^b^-SIINFEKL, the fraction of polyclonal anti-VACV CD8^+^ T cells that can be stimulated by infected cells should rise over time with a simple, rapid kinetic. If on the other hand, new pMHC complexes first appear on the cell surface at different times after infection, then one might expect a more complicated curve as new populations of T cells are able to be activated once their epitope appears at the cell surface. Thus using DC2.4 and the same infection protocol, global VACV epitope presentation was probed up to 12 hpi using splenocytes taken from mice seven days after VACV infection and the percent of CD8^+^ T cells making IFNγ determined by intracellular cytokine staining (ICS) (Figure 1B). In contrast to the simple rise of K^b^-SIINFEKL presentation, the increase in number of CD8^+^ T cells recognising the infected cells was more complex. There were two phases of rising CD8^+^ T cell activation, one from 2 to 5 hours (a similar time frame to K^b^-SIINFEKL presentation) followed by second, steeper rise from 5–7 hpi that continued until 12 hpi. While this reveals nothing about the kinetics of individual epitopes, it suggests that the onset of presentation differs across the native VACV epitopes. It is also consistent with published work using mono-specific T cell lines that shows presentation of some VACV epitopes is delayed for some hours after infection [35]. Together these data suggest that monitoring a single epitope does not reveal the true complexity of viral antigen presentation to T cells. We therefore sought to dissect in greater detail the presentation of individual VACV derived epitopes using mass spectrometry (MS).

### LC-MRM for the simultaneous detection and quantitation of multiple VACV epitopes

**Figure 2:**
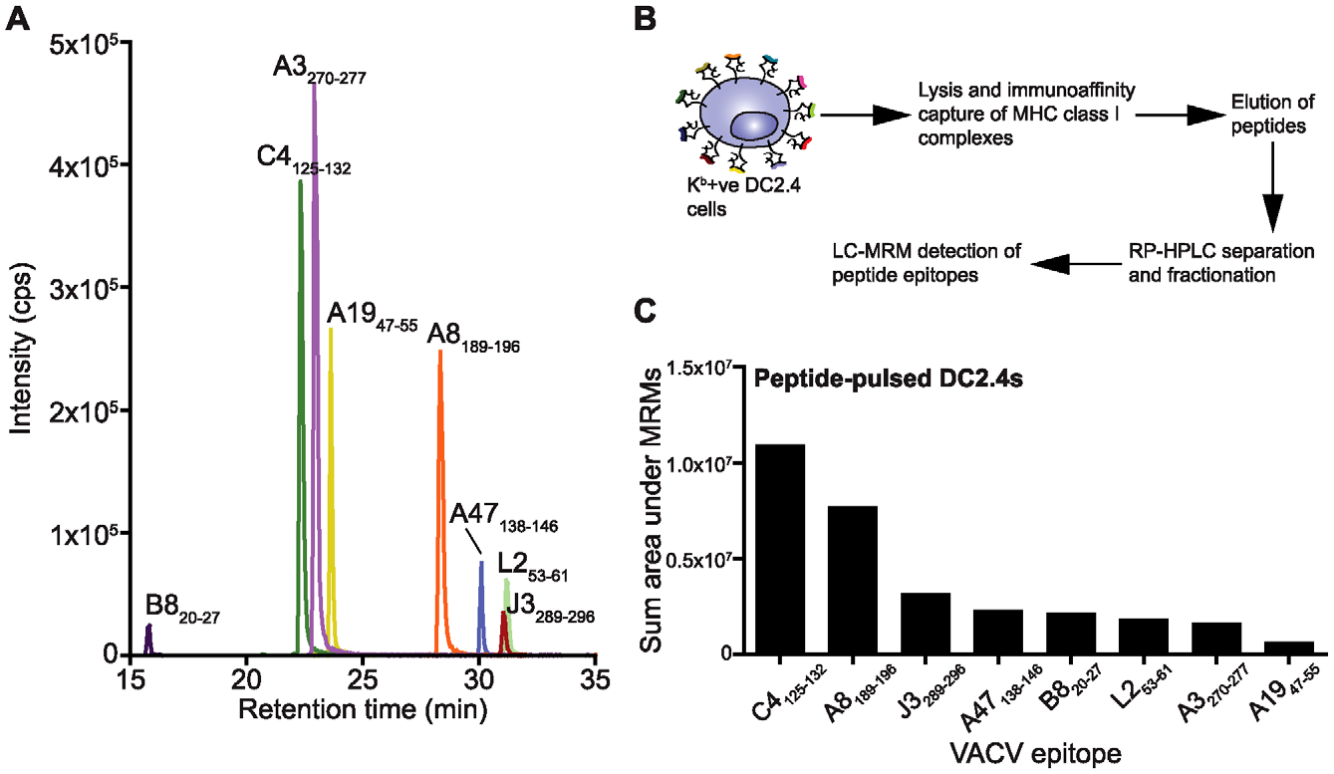
Simultaneous detection of multiple viral epitopes by LC-MRM MS. A) Demonstration of multiplexed detection of 8 VACV K^b^-binding epitopes. A mixture of 100 fmol of each synthetic peptide was loaded and analysed directly by LC-MRM using a method to detect all peptides simultaneously. A single MRM transition per peptide is shown for clarity. B) Schematic of sample workflow. C) DC2.4 cells were pulsed with a 1 µM mixture of each VACV peptide, incubated for 1 hour and washed extensively to remove unbound peptide. Cells were subjected to MHC-peptide elution and each epitope detected by LC-MRM-MS (sum area of all MRM transitions per peptide is shown).

Liquid chromatography coupled to multiple reaction monitoring mass spectrometry (LC-MRM) is the method of choice for detection of multiple known peptides [32], [36], [37]. LC-MRM MS affords high sensitivity and selectivity and has been recently applied to multiplexed qualitative and quantitative analyses of peptide epitopes eluted from MHC molecules [32], [37]. For this study, eight VACV epitopes restricted by murine H-2 K^b^ were chosen based on their well characterised immunogenicity and their expression from a variety of different VACV proteins spanning different temporal phases of the infection (Table 1) [4], [5]. In addition, SIINFEKL was included in some experiments to allow a direct comparison of this model antigen with the native VACV epitopes. Optimal MRM transition conditions (precursor ion charge, fragmentation energy and fragment ion selection) for each VACV epitope listed in Table 1 were determined using synthetic peptides (Table S1 and Figure S1 in Supporting Information). The resulting MRM method allowed for the simultaneous detection of all 8 VACV epitopes (Figure 2A) and also included transitions to measure SIINFEKL and isotopically-labelled (AQUA) SIIN*FEKL; inclusion of the SIIN*FEKL AQUA peptide was used to control for losses during processing of the MHC-bound peptides as described [32]. The unequivocal detection of peptide epitopes was achieved by several rigorous confirmatory steps in this LC-MRM workflow: firstly, RP-HPLC retention across multiple dimensions of purification (correct eluting fraction during off-line RP-HPLC and correct on-line retention time during LC-MRM MS) must be consistent with that measured for the synthetic version of each of the VACV peptides (Figure S2); secondly, they must trigger all MRM transitions concurrently and in the correct transition hierarchy; and, as a final step, each peptide sequence must be further confirmed by an MRM-triggered MS/MS sequencing scan – a modality unique to the quadrupole linear ion trap mass spectrometer used in this study [38].

**Table 1:** VACV CD8^+^ T cell epitopes.

Epitope	Sequence	IC_50_ (nM)	Protein Function	Expression*	Promoter**
B8_20–27_	TSYKFESV	K^b^, 1.1	Immune modulator	E1.1	E
A8_189–196_	ITYRFYLI	K^b^, 6.2	Viral transcription factor	E1.1	E
A3_270–277_	KSYNYMLL	K^b^, 2.7	Virion core protein	PR	I
C4_125–132_	LNFRFENV	K^b^, 0.84	Unknown	E1.2	E
A47_138–146_	AAFEFINSL	K^b^, 0.61	Unknown	E1.2	E
L2_53–61_	VIYIFTVRL	K^b^, 0.85	Unknown	E1.1	E
J3_289–296_	SIFRFLNI	K^b^, 1.2	Viral RNA pol component	E1.2	E
A19_47–55_	VSLDYINTM	K^b^, 0.94	Unknown	PR	I

* [41] Expression time based on cluster analysis of transcript levels during infection. E1.1 and E1.2 are early classes, with E1.1 expressed earlier and at higher levels that E1.2. PR (post replication), typically referred to as the late class.

** [43] Earliest promoter associated with gene. E is early, I is intermediate.

### Validation of LC-MRM and detection of VACV epitopes eluted from cells

In order to verify the sample workflow (Figure 2B), DC2.4 cells were incubated with a pooled mixture of the full set of 8 synthetic peptides representing VACV epitopes (Table 1). Following extensive washing to remove unbound peptides, cells were pelleted and snap-frozen and subjected to immunoaffinity purification of H-2K^b^ complexes, peptide elution and chromatographic separation as previously described [32], [37]. The presence of each VACV epitope in the MHC eluate was confirmed by LC-MRM (Figure 2C). The differing detection intensities across the peptide set reflects a combination of the varying ionisation efficiencies of the peptides and competition for binding to the K^b^ molecules during incubation.

**Figure 3:**
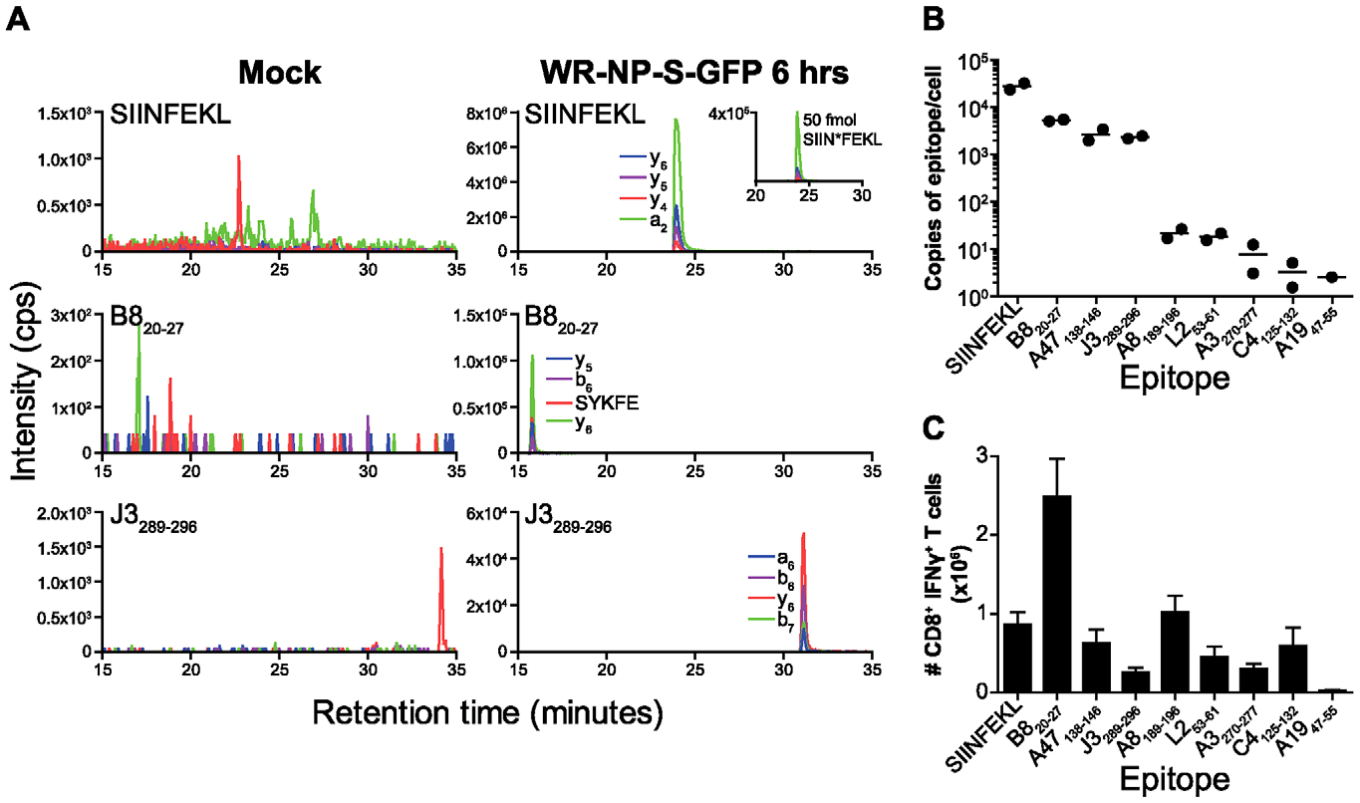
Detection and quantitation of SIINFEKL and VACV epitopes from WR-NP-S-GFP infection. 1×10^8^ DC2.4 cells were either mock treated or infected with 5 pfu of VACV strain WR-NP-S-GFP for 6 hours and epitopes eluted and analysed by the LC-MRM workflow. A) Representative MRM traces for the detection of SIINFEKL (RP-HPLC fraction A10) and VACV epitopes B8_20–27_ (fraction A8) and J3_289–296_ (fraction A13). Background, non-specific MRM signal is shown for mock treatment (left panels). Positive, overlapping MRM transitions (four per peptide, Q3 ions as indicated) are shown during infection (right panels). Inset: MRM signal for 50 fmol of the internal standard isotopically-labelled AQUA peptide SIIN*FEKL for the absolute quantitation of SIINFEKL levels. B) Epitope presentation hierarchy on DC2.4 cells 6 hours after infection with WR-NP-S-GFP by LC-MRM. Data from 2 independent infections are shown. C) CD8^+^ T cell immunodominance hierarchy 7 days after infection of C57BL/6 mice as determined by brief stimulation with the peptides shown and ICS for IFNγ. Data are the mean and SEM of 3 mice and are representative of two experiments.

Next, MHC elution and LC-MRM were used for the detection of SIINFEKL and native VACV epitopes generated through VACV infection with the recombinant WR-NP-S-GFP. DC2.4 cells (1×10^8^) were infected for 6 hours with WR-NP-S-GFP to compare the levels of SIINFEKL presentation with that of the 8 native VACV epitopes (Figure 3). Capture of K^b^-peptide complexes was achieved as above, including the addition of 50 fmol of isotopically-labelled AQUA SIIN*FEKL in order to control for sample preparation losses post affinity purification of the MHC-peptide complexes [32]. The quantification of each VACV epitope was achieved by comparing the area under the MRM curve to that of 100 fmol of the corresponding synthetic epitope analysed separately (Figure 2A). LC-MRM confirmed the detection of SIINFEKL and all 8 VACV peptides (Figure 3A shows representative data for SIINFEKL, B8_20–27_ and J3_289–296_). Further it provides the first definitive evidence that the amino acid length and constitution of the VACV epitopes is exactly as described in the original mapping studies [4], [5]. SIINFEKL presentation on WR-NP-S-GFP-infected cells at 6 hpi was calculated to be 2.3×10^4^ and 3.1×10^4^ copies per cell for two independent experiments (Figure 3B). All 8 K^b^-restricted VACV epitopes were detected at considerably lower estimated abundances to that of SIINFEKL. Further, abundance of the 8 VACV peptides varied over a wide range with 3 epitopes (B8_20–27_; A47_138–146_ and J3_289–296_) being presented at levels up to 1000-fold higher than the remaining 5 VACV epitopes. When compared to CD8^+^ T cell response elicited in mice infected for 7 days by the same virus, there is a striking dissociation between the epitope abundance and T cell immunodominance hierarchies (Figure 3B).

### Kinetics of VACV epitope and protein presentation

**Figure 4:**
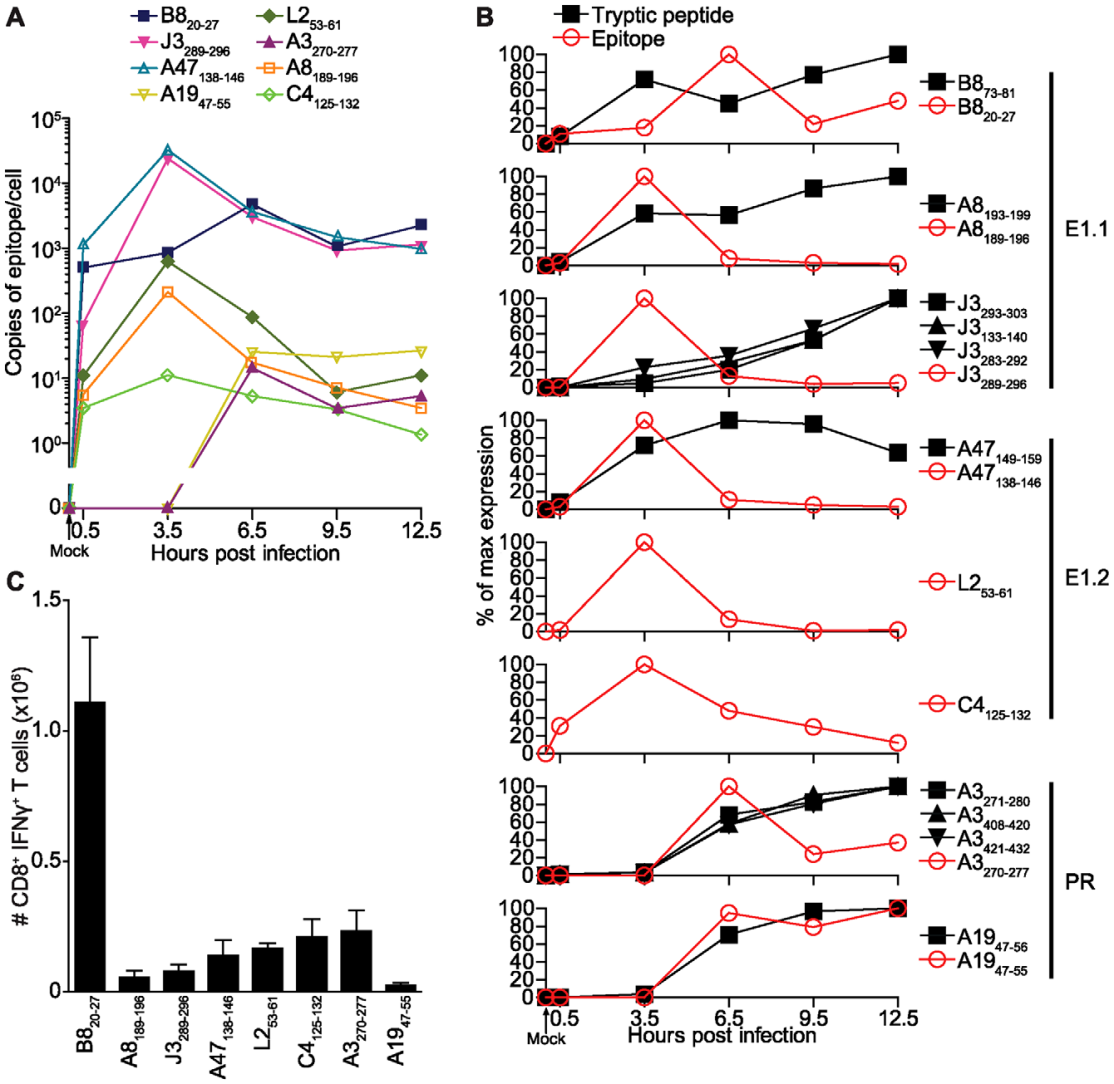
Kinetics of VACV antigen presentation. A) 1×10^8^ DC2.4 cells/time point were infected with 5 pfu of VACV strain WR (or mock treated as time 0) and incubated for 0.5, 3.5, 6.5, 9.5 or 12.5 hours. MHC-peptide complexes were eluted at each time and epitope levels monitored by LC-MRM. Data show the copies of each epitope per cell. B) Epitope data from (A) expressed as the percentage of maximum levels alongside the relative kinetics of each source protein. C) CD8^+^ T cell immunodominance hierarchy of WR infection 7 days after infection as determined brief stimulation with the peptides shown and ICS for IFNγ. Data are the mean and SEM of 3 mice and are representative of multiple experiments.

Next we sought to assess the presentation kinetics of the 8 VACV epitopes during the course of infection. This was done using non-recombinant VACV, to avoid any potential competing effects from the very high levels of presentation of SIINFEKL following infection with the recombinant WR-NP-S-GFP VACV strain. DC2.4 cells were infected for 0.5, 3.5, 6.5, 9.5 and 12.5 hours, or mock infected as a negative control and epitope abundance at each time determined by LC-MRM analysis. All 8 VACV epitopes were detected and the kinetics of their presentation measured (Figure 4A). Six of 8 peptides were detected by 0.5 hpi, with the remaining 2 epitopes (A3_270–277_ and A19_47–55_) undetectable until 6 hours later. Peak expression occurred at 3.5 hpi for 5 epitopes, 6.5 hpi for two epitopes and at the final time point of 12.5 hours for a single epitope. We noted that the presentation of the immunodominant B8_20–27_ epitope was unusual in that its onset was at 30 minutes, but instead of peaking at 3.5 hpi, like most of this group of epitopes, its peak was later at 6.5 hpi. The abundance profile spanned 3 logs, ranging from as low as an estimated 11 copies per cell for C4_125–132_ to as high as 32,400 copies of A47_138_. These basic features of presentation with some epitopes showing peak presentation around 3.5 hours after infection, while others only appear at 6.5 hours have also been observed for cells infected with the MVA strain of VACV (our unpublished observations). Thus abundance and kinetics of presentation are highly variable across different epitopes and robust presentation early after infection is not always maintained.

In order to assess how the kinetics of epitope presentation correlates with source antigen expression, a sample of the cell lysate from each infection time point was subjected to reduction, alkylation and subsequent digestion with the enzyme trypsin prior to proteomic analysis. Proteotypic tryptic fragments from each of the 8 VACV protein antigens were chosen using Skyline [39] (Table S2 and Figures S3 and S4). Following initial screening of samples, 6 of the 8 VACV proteins were detected (for A3 and J3, multiple tryptic fragments were found to be amenable to MRM analysis and so all were included). Despite rigorous testing of multiple peptides, no positive signal could be detected for proteins L2 and C4 so these were not included further. In order to achieve normalisation of protein loading across the timecourse, 12 murine tryptic peptides (corresponding to eight host proteins; Table S3 and Figure S3) were simultaneously analysed in the same LC-MRM method (Figure S3). These murine proteins were chosen as suitable candidates for normalisation based on the high copy number and long half life of their human homologues [40], with the notion that such proteins will not be grossly affected by the VACV-mediated shutdown of host protein synthesis. In addition, a good correlation between the abundance of these representative proteins and cell number recovered post-infection was found suggesting that they were appropriate for normalisation (Figure 3C). The uncorrected data is also shown in Figure S4 for comparison.

MRM peaks at each time point for the 6 VACV proteins were used to determine relative protein expression over the course of infection and these were plotted alongside the relative levels of each epitope derived from the same protein (Figure 4B). This approach allows relative expression of individual antigens to be determined at different time points but does not provide absolute quantitation of the antigen and therefore direct comparison between antigens is more qualitative. Expression profiles of the 6 proteins were consistent with their temporal expression cluster as reported by analyses of transcription and more recently defined promoters [41]–[43], which gives further confidence of the method. Translation, as determined by tryptic peptide detection, was detected at 0.5 hpi for A47, A8 and B8, corresponding with the appearance of epitopes derived from those proteins. Whilst levels of A47 peaked at 6.5 hpi, all other proteins peaked (at least within the limits of this time course) at 12.5 hours. Proteins A3 and A19, both of which are classified as late, were detected by 3.5 hours, but did not reach substantial levels until 6.5 hours and onwards; presentation of epitopes A3_270–277_ and A19_47–55_ tracked closely with the increase in protein levels. For epitopes A47_138–145_, A8_189–196_ and J3_125–132_, rapid and peak presentation following protein expression was followed by a sharp decline in epitope levels to almost zero by 12.5 hpi. However, epitopes B8_20–27_ and A3_270–277_, although decreasing following peak levels mid-infection, maintained a more constant level around 20–40% of the maximum; for A19_47–55_, epitopes levels did not peak until the end of the time course, following an almost identical profile to A19 protein expression. Of note the B8_20–27_ epitope appeared to display a lag between peak of protein expression and peak of epitope presentation.

Next, *in vitro* protein and epitope presentation kinetics were correlated with CD8^+^ T cell immunodominance *in vivo*. C57BL/6 mice were infected with VACV WR by the intraperitoneal route (i.p.) and 7 days after infection, the percentage of CD8^+^ T cells responding to *ex vivo* stimulation with each peptide was determined by intracellular staining for IFNγ (Figure 4C). This method of epitope detection has recently been shown to have a linear range that covers responses to all the epitopes investigated here [44]. As previously reported [4], [5], B8_20–27_ dominated the response, A19_47–55_ was the weakest and the remaining 6 epitopes formed an intermediate hierarchy. Here, where the onset, peak level and longevity of epitope display were revealed (as opposed to the single time point for the WR-NP-S-GFP in Figure 3), there was still no obvious correlation between presentation and the CD8^+^ T cell dominance hierarchy. Although the immunodominant B8_20–27_ was one of the most robust epitopes in peak and persistence of presentation, it is similar in this respect to the subdominant A47_138–146_ and J3_289–296_. Further, A3_270–227_ and A8_189–196_, which are the next 2 peptides in the dominance hierarchy after B8_20–27_, have very different presentation profiles with the former only appearing later (6.5 hpi) and having better persistence but a substantially lower (approximately 10-fold) peak than the latter.

## Discussion

The use of liquid chromatography and mass spectrometry to detect MHC epitopes has a long heritage [e.g. [45]–[50]] yet it is only in recent years that techniques and instrumentation are beginning to surpass sensitivity and feasibility blockades to gain qualitative and quantitative insights into the immunopeptidome [24], [32], [51]–[53]. Use of LC-MRM methods to detect epitope presentation offers a large increase in sensitivity, but thus far has few precedents in the literature. LC-MRM analysis has rarely been used to examine antigen presentation with only a few examples examining melanoma epitopes [54] and measles virus epitopes [55]. We have recently further developed the methodology studying SIINFEKL presentation as a model antigen [32]. The current study is the first to comprehensively apply LC-MRM to study epitope presentation during virus infection, an inherently dynamic process. It is also the first to combine epitope and source antigen quantification from the same samples using LC-MRM. Our data provide extensions to and have implication for several aspects of antigen processing and anti-viral immunity and include:

### 

#### 1. Confirmation of epitopes mapped by prediction and synthetic peptides

The variable quality of epitope mapping data is a problem that is too frequently ignored [56], [57]. Of the 8 epitopes examined here, 3 have been verified to the level of the source antigen and by peptide titration [4]. For the remaining 5, the only data available are from prediction and testing of peptides *in vitro* at relatively high concentration [5]. The nature of the MRM method leaves no ambiguity as to the sequence of peptides that are detected on infected cells and all 8 peptides have now been confirmed as the actual epitope presented on the surface of infected cells. Our data supports the value of the predictions and methods used to map such epitopes using overlapping synthetic peptides; but a wider comparison of the many epitopes published from a variety of viruses and other pathogens remain an important future goal.

#### 2. Complexity of epitope kinetics

**Figure 5:**
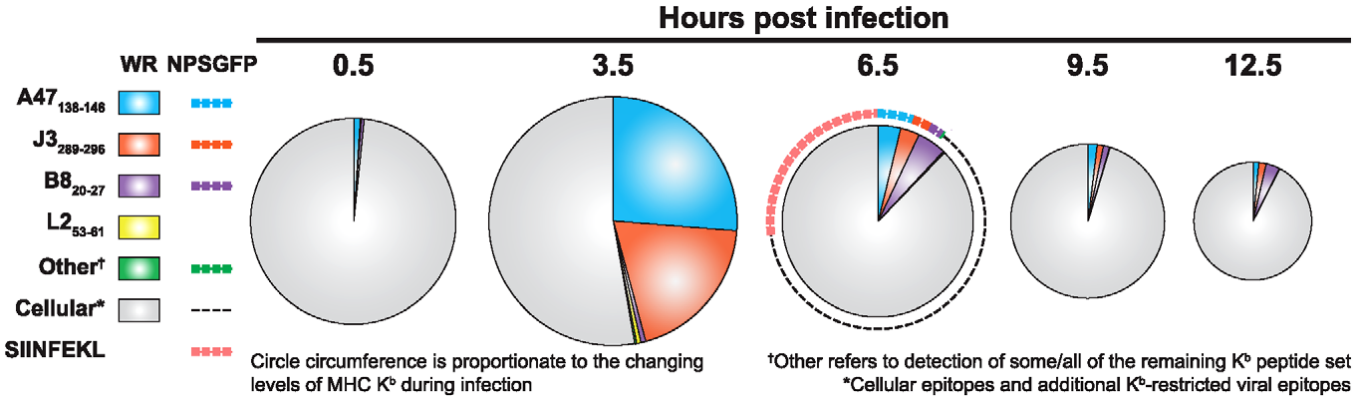
Cartoon depiction of the changes in vaccinia viral epitope presentation during infection. Circles represent infected cells at the indicated times post infection. Size is proportionate to the changing levels of MHC K^b^ during infection, relative to mock-infected cells.

We show here that the abundance of virus epitopes varies massively from as low as 10 copies per cell to as high as >30,000 copies per cell. This variation is striking given that these are all relatively immunogenic peptides and responses to 6 of 8 epitopes fall within a two-fold range when measured using *ex vivo* assays (Figures 3C and 4C). The kinetics of epitope presentation was much more complex than that observed for single epitopes; not all epitopes were presented by 3.5 hours but all were there at 6.5 hpi consistent with data produced with mono-specific T cell lines [35]. Moreover, the dominant B8_20–27_ epitope peaked at the 6.5 h time point, consistent with the rise in antigenicity seen on the infected cells between 5 and 7 hours (Figure 1B). To provide another view of epitope presentation, we have expressed our data in terms of the fraction of surface H-2K^b^ occupied by the epitopes studied (Figure 5). To do this we have assumed a basal copy number for this MHC allomorph of 10^5^ class I copies per cell, which although is likely to be conservative, is in line with published estimates [48], [58]. Moreover, we have taken into account variation in cell surface MHC levels during the infection time course based on data collected under identical experimental conditions (Figure S5). This analysis highlights several striking features: First, as early as 3.5 hpi a very large proportion of all H-2K^b^ on the infected cells are displaying virus-derived epitopes, which represents a very rapid and substantial change from the pre-infection immunopeptidome. This also contrasts with HIV infection, where the most noteworthy change after infection was the presentation of novel host epitopes [16], suggesting that different viruses will have different impacts upon the immunopeptidome and thus may explain the very different evolution of immune responses to some viruses. Second, antigen presentation can be dominated by relatively few epitopes. At 3.5 hours after infection, presentation of just 2 virus epitopes (A47_138–146_ and J3_289–296_) can account for an estimated half of all H-2K^b^ molecules. This domination of presentation by few virus epitopes has precedents, but has not been demonstrated so soon after infection [17]. Third, presentation is very dynamic, exemplified by the rise and fall of A47_138–146_ and J3_289–296_ from 0.5 to 6.5 hours. The rapid loss of presentation of these epitopes is intriguing because they both have relatively high affinity for H-2K^b^ molecules (Table 1: IC_50_ of 0.61 and 1.2, respectively [59] (although our own data suggests J3_289–296_ stabilises K^b^ poorly) and in each case the source protein continues to accumulate over this time. The loss of these peptides is not easy to reconcile with half life predictions for H-2K^b^ ranging from a lower limit of around 48 minutes for empty forms [60], to estimates with peptides of moderate to high affinity of over 100 minutes [61]. However, half life is determined by the balance of association and dissociation rates [62], which are not fully known for these peptides. In our own studies (Figure S6), we find that a peptide's capacity for increased MHC stabilisation correlates roughly with the half life of that complex on the cell surface, yet neither of these factors can predict the kinetics observed in our study. This is perhaps not surprising given that such measured half lives are for cells in a steady state condition and not undergoing virus infection. As a virus such as VACV takes over all aspects of cell biology the supply of peptides will change abruptly. Just as virus epitopes rapidly replace those from the host, swamping the presentation capacity of cells at 3.5 hours, it is reasonable to suggest that the cascade of gene expression during infection is mirrored by successive waves of virus epitopes being presented. A final feature of Figure 5 is the comparison of data from cells infected with WR and WR-NP-S-GFP at the 6.5 hour time point. This shows very similar amounts of the native virus epitopes for both viruses, strongly supporting the validity of our normalization methodology. Whilst it also shows that SIINFEKL is more prevalent than any of the VACV epitopes in our study at 6.5 hours, compared with the H-2K^b^ occupancy levels achieved by A47_138–146_ and J3_289–296_ at 3.5 hours it is by no means exceptional. Moreover, like A47_138–146_ and J3_289–296_, SIINFEKL is not as immunogenic as the B8_20–27_ epitope.

#### 3. Relationship between antigen and epitope levels

Previous studies have used various strategies to relate antigen expression or steady state levels to explain epitope abundance. On balance, studies comparing transcription and epitope levels show that the correlation between transcript levels and epitopes presented is not especially good [24], [28]. Likewise, a proteomic approach using stable isotope labelling with amino acids in tissue culture (SILAC), found a limited correlation between the proteome and immunopeptidome [63]. A dynamic setting in which antigen levels are changing can provide more insight into the relationship between antigen and epitope levels. An example of this approach found that novel host cell epitopes appearing on HIV-infected cells was most likely related to the degradation of these proteins during infection and not level of expression [16]. In contrast, here we focus on virus antigens and epitopes and are able to correlate protein levels from the initiation of their synthesis. What is most clear from our study is that onset of epitope presentation is essentially coincident with neosynthesis of the source proteins. For 4 of the 6 epitopes where protein data were obtained, epitope abundance peaks before antigen abundance; for A19, epitope and protein kinetics were essentially coincident, and for the remaining epitope (B8_20–27_) protein abundance was found to peak first. Further, as noted above epitope turnover after the peak is often rapid, in contrast to the source proteins that typically continue to slowly rise or plateau. These data also demonstrate the power of the time course approach in that without the full kinetics of epitope and antigen levels, the conclusions would depend entirely on the time point chosen for analysis. The inescapable conclusion from our data is that epitope presentation is not well correlated with antigen abundance, but this is in part because of the rapid proteome dynamics in virus infected cells. The caveat here is that in our analysis we were not able to determine absolute protein abundance and so we cannot draw conclusions across the epitopes.

#### 4. The source of virus epitopes

For many model antigens neo-synthesis of proteins and not their eventual degradation correlates with epitope production [25]–[27], [64]. The rapid onset of epitope presentation that coincides with antigen expression in our study, strongly support this idea. Importantly we show this close coupling of expression and presentation for each of the 6 epitopes and these are derived from native virus proteins expressed in the normal course of infection. This means first, that the linkage of expression and presentation is indeed a general phenomenon. Second, that any concerns about previous findings being linked to the artificial nature of the model antigens or systems being used can now be discarded. What remains is to clearly reveal the identity of the source proteins: what fraction of these are the defective products predicted by the DRiP hypothesis versus other forms of rapidly degraded proteins, perhaps produced by the pioneer round of mRNA translation [65], [66]. Either way, the efficiency implied by the appearance of 6 of our 8 epitopes as early as 30 minutes after infection suggests an exceptionally tight relationship between a subset of newly synthesised proteins and the antigen processing machinery. On the other hand, the presentation of post-translationally modified antigens in other studies presumably reflects that degradation of more mature protein species does contribute to the epitopes presented [67], [68]. A full understanding of the fraction of epitopes derived from newly synthesized versus mature proteins awaits a full immunopeptidome-wide analysis. The behaviour of B8_20–27_ is of interest in this respect: while onset of protein production and presentation were co-incident, peak presentation was delayed. We speculate that this antigen is processed by two pathways, with epitope being produced both from nascent protein, but also from a pool of mature protein. So while we show here the benefits of broad-scale approaches in allowing the common themes to emerge, clearly much remains to be learnt from detailed analyses of individual antigens.

#### 5. Antigenicity on infected cells and immunogenicity

In our experiments with both VACV viruses there is no correlation between levels of epitope display on infected cells and immunogenicity. Others have shown that epitope levels measured at a single time are not helpful in predicting the immunodominance hierarchy beyond suggesting that very low epitope levels can limit immunogenicity [47], [69]. However, rate of epitope accumulation [69] and epitope off rates [70] or direct measurements of persistence [71] have all been suggested to be important determinants of immunogenicity. For the first time we are able to determine onset, peak and persistence of presentation for multiple epitopes and show that none of these parameters were useful indicators of immunogenicity. There are two main areas of explanation here. First we are using a cultured cell type that may or may not resemble the real APC for priming anti-VACV CD8^+^ T cells. The balance of more recent studies suggests that during VACV infection most CD8^+^ T cells are directly primed by infected DCs [29]–[31], but it remains possible that some epitopes may utilize cross priming [72]. If indeed cross priming is important, levels of epitope on cross priming DCs would almost certainly be different than those on infected cells and this might explain the discrepancy between our data on presentation and immunogenicity. Even if direct priming is the main pathway, numerous possible complications in presentation might be in play. More than one APC type might be involved and these might present varying levels of individual epitopes through an array of mechanisms. For example, differential immunoproteasome expression [73], [74] or susceptibility to viral immune evasion mechanisms [75]. Infection might be aborted in some APCs and depending on the time that this occurs, epitopes from later classes of genes would not be presented. Of interest in this regard is that of the nearly 50 H-2^b^-restricted epitopes mapped, only two very weak ones (far weaker than A19L examined here) are from genes with late promoters [5], [43]. Further, epitope display on non-professional APCs might contribute to expansion of some specificities after priming [76]. It will be important to repeat our studies by harvesting APC directly *ex vivo* which will take into account epitopes acquired by any mechanism. Second, immunodominance is a complicated business and is driven by factors other than availability of antigen. Precursor frequency for responding CD8^+^ T cells will always be a critical part of the explanation [77], [78]. However, studies of VACV and influenza virus suggest that even epitope abundance and the T cell repertoire might not be enough to fully explain immunodominance [79], [80]. Thus much work remains both on the antigen presentation and T cell sides of the immunodominance equation.

#### 6. The value of individual epitopes as CD8^+^ T cell vaccine targets

The sharp peak of epitope presentation kinetics and delayed appearance of some epitopes are important factors to consider when using epitope abundance data to choose optimal vaccine targets. These considerations again highlight the importance of measuring presentation at multiple time points. Having said this, despite the vast difference in presentation levels, all the peptides in our study were shown to be highly protective (>70% survival) against a lethal VACV challenge when used as a sole immunogen [59]. As noted above, better studies to relate the protective capacity of epitopes with their display on infected cells will be subject to identifying and studying the relevant cell type *in vivo*.

In summary, the advances we present here in quantifying virus epitope abundance set a new benchmark in our understanding of antigen presentation during virus infection. The surprisingly dynamic nature of epitope display is a feature that has not been reported previously and has ramifications both for immunogenicity and use of epitopes as targets. This study is an important step toward the ultimate goal of quantifying epitope presentation *in vivo* which in turn is a requisite for a full understanding of anti-viral T cell responses and how they may be manipulated in future vaccines and other immunotherapies. Finally, the capacity to follow viral protein expression, induced host cell protein expression and antigen presentation will provide new avenues of research into the virus-host interaction and the role of immunoevasins and innate immune mechanisms in viral clearance.

### Materials and Methods

#### Cell lines

The murine bone marrow-derived DC line DC2.4 [81] was provided as a kind gift from Professor Kenneth Rock (University of Massachusetts Medical School). The murine hybridoma Y-3 [82] secretes an anti-H-2K^b^ monoclonal antibody and RMA-S is a TAP-deficient cell line derived from C57BL/6 mice [83]. Cells were maintained in RPMI or DMEM (Life Technologies) supplemented with 10% fetal bovine serum (FBS), 2 mM glutamine with or without 50 IU/ml penicillin and 50 µg/ml streptomycin (R10 or D10). BHK-21 and BS-C-1 were maintained in D10.

#### Virus production

VACV strains Western Reserve (VACV WR, ATCC #VR1354) and WR-NP-S-GFP were grown and titrated in BHK-21 and BS-C-1 cells respectively under DMEM with 2% FBS and 2 mM glutamine (D2) using standard methods. VACV WR and WR-NP-S-GFP were gifts of Bernard Moss, Jon Yewdell and Jack Bennink (all at NIH, Bethesda).

#### Virus infection

For LC-MRM experiments DC2.4 were washed twice with DMEM with no additions (D0), then 1×10^8^ cells were infected at 10 plaque forming units (pfu) per cell in 2 ml D0 in round-bottom tubes for 30 minutes at 37°C with shaking. After this time the cells were transferred to 50 ml tubes and 40 ml of warm D2 was added. The tubes were then incubated for the required time at 37°C with slow rotation. Cells were counted at the end of each incubation to account for any loss due to the method and snap frozen. For detection of K^b^-SIINFEKL and for use as stimulators prior to ICS, 1–5×10^6^ of washed DC2.4 were infected with 10 pfu/cell of WR or WR-NP-S-GFP in 0.2 ml of D0 for 60 minutes at 37°C with shaking. After this time 10 ml of warm D2 was added and the tubes were incubated for the required time at 37°C and then placed on ice before staining with mAb 25D1.16 or use in intracellular cytokine staining (ICS).

#### Peptide synthesis

Peptides (>80% purity) were purchased from Genscript Corp (Piscataway, NJ) or Mimotopes (Clayton, Vic, Australia) and master stocks made with 100% DMSO at 1 mg/ml or greater. Dilutions were made in D0. For mass spectrometry, purified and lyophilised peptides were reconstituted in 100% DMSO at 5 mM and diluted with buffer A (0.1% formic acid in water) to stocks of 2.5 µM.

#### Flow cytometric staining of K^b^-SIINFEKL levels

DC2.4 cells (1×10^6^) infected with WR-NP-S-GFP were incubated with mAb 25D1.16 conjugated to Allophycocyanin (eBioscience) [8], [12] for 30 minutes on ice. Cells were washed and analysed by flow cytometry (LSR II, BD Biosciences) and analysis done using Flowjo software (Tree Star).

#### Stimulation and intracellular cytokine staining (ICS)

Splenocytes (1×10^6^) from mice infected 7 days previously with VACV WR or WR-NP-S-GFP were incubated with 1) peptides at 10^−7^ M or 2) 2×10^5^ DC2.4 cells infected with VACV (see above) in the wells of a 96-well plate at 37°C and 5% CO_2_. 10 µg/ml brefeldin A was added after 1 hour, and the incubation continued for another 3–4 h. Plates were spun, medium was removed, and cells were stained for surface CD8 (clone 53–67, BD Biosciences) on ice for 20 minutes. Cells were washed, fixed with 1% paraformaldehyde solution, washed and then stained for intracellular IFNγ (clone XMG1.2, BD Biosciences) in PBS with 0.5% saponin at 4°C. Cells were washed and analysed by flow cytometry (LSR II, BD Biosciences) and analysis done using Flowjo software (Tree Star). Background staining determined using uninfected cells (generally around 0.1%) was subtracted from the values presented from infected samples. The fidelity of this method for enumeration of CD8^+^ T cell responses to acute VACV infection is supported by a recent study [44].

#### Purification of MHC-peptide complexes

Frozen cell pellets of DC2.4 were lysed by gentle resuspension in a total of 5 mls of 0.5% IGEPAL (Sigma), 50 mM Tris pH 8, 150 mM NaCl and protease inhibitors (Complete Protease Inhibitor Cocktail Tablet; Roche Molecular Biochemicals) and incubated with rotation for 1 hour at 4°C. Lysates were cleared by centrifugation at 16,000×g in a benchtop microfuge and MHC-peptide complexes immunoaffinity purified using Y-3 (anti-K^b^) monoclonal antibody bound to protein A-Sepharose, as previously described [32], [84]. Bound complexes were eluted by acidification with 10% acetic acid. The mixture of peptides and MHC protein chains was fractionated on a 4.6 mm internal diameter×50 mm long reversed-phase C18 HPLC column (Chromolith Speed Rod, Merck) using an ÄKTAmicro HPLC system (GE Healthcare) running on a mobile phase buffer A of 0.1% trifluoroacetic acid (TFA) and buffer B of 80% acetonitrile/0.1% TFA and at a flow rate of 1 ml/min with peptides separated across a gradient of 2% B to 45% B over the course of 20 minutes, collecting 500 µl fractions.

#### FASP protein purification and tryptic digestion

Lysate flow-through following immunoaffinity purification was used to detect cellular and VACV proteins. 200 µl of flow-through was treated with the reducing agent (*tris*(2-carboxyethyl)phosphine (TCEP) at a final concentration of 5 mM and sample heated to 60°C for 30 minutes. 30 µl of sample was then loaded onto a FASP protein digestion kit column (Protein Discovery) [33] and tryptic digestion of proteins carried out as per the manufacturer's instructions. Digested proteins were eluted from the column with 50 µl of 0.5 M sodium chloride into 80 µl of 50 mM ammonium bicarbonate. 10-fold dilutions of the eluate (starting from 10 µl neat, to a 1/1000 dilution) were made to a 20 µl volume with mass spectrometry buffer A (0.1% TFA in water) and analysed by LC-MRM as described below (see *Liquid chromatography mass spectrometry*).

#### Design of VACV MRMs

MRM transitions for 8 VACV epitopes, restricted through murine K^b^ MHC class I molecules, were designed through spiking 200 fmol of synthetic versions of each peptide into an AB-SCIEX QTRAP 5500 mass spectrometer (Table S1 and Figure S1). Each peptide was initially analysed in EMS scanning mode to determine the predominant precursor (Q1) ion. Peptides were then analysed in EPI scanning mode across a range of collision energies (CEs) to determine the optimal CE resulting in the highest intensity fragment ions in Q3. At least four Q3 ions were chosen per peptide to eliminate isobaric peptides triggering false-positive MRMs, and each Q3 ion was fine-tuned for optimal intensity using the method of Sherwood *et al* [*85*]. All MRMs were built into a single method which also included the previously described MRMs for SIINFEKL and isotopically-labelled (AQUA) SIIN*FEKL [32].

#### Design of theoretical MRMs for VACV and murine protein antigen detection

Skyline [39] v1.2.0.3245 was used to build an initial library of MRMs targeting proteotypic tryptic fragments for each VACV protein from which epitopes in this study were derived (Table S2 and Figure S4). Up to 3 tryptic fragments were selected per protein, with at least 3 transitions per peptide. MRMs for each peptide were further refined upon their detection. 12 tryptic murine peptides (corresponding to 8 proteins) were used to normalise protein levels across the timecourse (Table S3 and Figure S3).

#### Liquid chromatography mass spectrometry

Following peptide elution, samples were concentrated using a Labconco Centrivac concentrator, set to 40°C. Samples were concentrated down to a volume of <10 µl and then all equalised to 20 µl through the addition of 0.1% formic acid in water (buffer A), sonicated in a water bath for 10 minutes and centrifuged for 10 minutes at 13,000 rpm prior to samples being stored in mass spectrometry vials at 4°C for immediate analysis by mass spectrometry. An AB SCIEX QTRAP 5500 mass spectrometer was used for MRM detection, coupled on-line to a Tempo nano LC (Eksigent) autosampler and cHiPLC nanoflex (Eksigent). 20 µl samples were injected and loaded onto a trap column (200 µm×0.5 mm ChromXP C18-CL 3 µm 120 Å; Eksigent part number 804-00006) at a flow rate of 10 µl/min in 98% buffer A for 10 minutes. For on-line fractionation of samples onto the mass spectrometer, samples were eluted from the trap column and over a cHiPLC column (75 µm×15 cm ChromXP C18-CL 3 µm 120 Å; Eksigent part number 804-00001) at 300 nl/min under the following buffer B (95% acetonitrile, 0.1% formic acid in water) gradient conditions: 0–3 min 2–10% B, 3–33 min 10–40% B, 33–36 min 40–80% B, 36–38 min hold at 80% B, 38–39 min 80–2% B, followed by equilibration at 2% B until the end of the run at 48 min. The QTRAP 5500 was operated in MRM mode in unit resolution for Q1 and Q3, coupled to an information-dependent acquisition (IDA) criterion set to trigger an EPI scan (10,000 Da/sec; rolling CE; unit resolution) following any MRM transition exceeding 500 counts (ignoring the triggering MRM transition for 3 seconds thereafter).

#### Mass spectrometry data analysis

Data analysis was performed using a combination of Analyst v1.5.2, Peakview v1.1 and Multiquant v2.0.2 (AB SCIEX).

#### RMA-S stabilisation assay

RMA-S cells were used to measure the capacity for each epitope to stabilise MHC K^b^ cells, as described previously [86]. Briefly, cells were grown overnight at 26°C to allow expression of peptide receptive class I molecules on the surface of cells. The following morning, decreasing concentrations of peptide were exogenously loaded onto the cells for 1 hour and then the cells transferred to 37°C for 2 hours to allow unloaded H-2K^b^ molecules to dissociate. Cells were then stained with the K^b^-specific monoclonal antibody Y-3, followed by secondary detection with a FITC-conjugated anti-murine IgG antibody (Merck Millipore; AQ326-K) and visualisation by flow cytometry. For measuring the time course of epitope stabilisation, cells were grown and labelled as above with 1 µM of peptide for 1 hour at 26°C, washed, and then incubated for the indicated amount of time. Class I molecules were detected as in the standard stabilisation assay. Data was analysed using FlowJo software (TreeStar).

#### MHC expression analysis during virus infection

DC2.4 cells (2×10^7^) were infected with VACV-WR at an m.o.i. of 5 in 500 µl of serum-free DMEM for 30 minutes. Infected cells were then transferred to 40 ml DMEM supplemented with 2% FCS. The tubes were then incubated for the required time at 37°C with slow rotation. Cells were centrifuged, incubated with Fc block (BD Biosciences; clone 2.4G2) for 20 minutes at 4°C. Cells were washed once and stained with PE-conjugated anti-H2-Kb (Biolegend; AF6-88.5) for 30 minutes at 4°C. Cells were wash and fixed with paraformaldehyde at room temperature for 20 minutes. Cells were washed and analysed by flow cytometry (LSR II, BD Biosciences) and analysed using FlowJo software (Tree Star).

#### Ethics

All experiments were done according to Australian NHMRC guidelines contained within the Australian Code of Practice for the Care and Use of Animals for Scientific Purposes and under approvals F-BMB-38.8 and A2011-01 from the Australian National University Animal Ethics and Experimentation Committee.

## Supporting Information

Figure S1 **MS and MS/MS analysis of synthetic VACV peptides for MRM design.** 200 fmol of each VACV peptide was analysed individually by a QTRAP 5500 operating in enhanced MS (EMS) mode in order to determine the dominant precursor ion (upper panel for each peptide; precursor ion charge is indicated). Subsequently, a full enhanced product ion (EPI) scan (80–1000 m/z range) across a range of collision energy (CE) values was triggered following detection of the dominant precursor (lower panel for each peptide; a single CE is shown for clarity, although different CEs were used for optimal product generation – refer to Table S1). Further refinement of optimal MRM conditions was achieved using a method adapted from Sherwood *et al.* (*Sherwood et al., 2009*). At least 4 Q3 product ions (purple lines) were chosen per peptide in order to practically eliminate false-positive signal due to the presence of isobaric peptide species in MHC eluates.
(TIF)

Figure S2 **Eluting RP-HPLC fractions for each epitope.** 1 nmol of each peptide was spiked individually onto a 4.6 mm internal diameter×50 mm long reversed-phase C18 HPLC column (Chromolith Speed Rod, Merck) using an ÄKTAmicro HPLC system (GE Healthcare) running on a mobile phase buffer A of 0.1% trifluoroacetic acid (TFA) and buffer B of 80% acetonitrile/0.1% TFA and at a flow rate of 1 ml/min. These conditions are identical to those used in the separation of peptides following MHC elution and therefore determines the RP-HPLC fraction (indicated above each chromatographic peak) which contains each VACV epitope. Peptides were read at an absorbance of 215 nm.
(TIF)

Figure S3 **The use of murine protein MRMs for the protein normalisation during VACV infection.** In order to accurately measure relative levels of VACV protein expression during infection (see Supporting Information Figure S4) it was necessary to concurrently measure host cellular protein levels as references for normalisation. Following protein lysate reduction and alkylation with iodoacetamide, levels of twelve tryptic peptides (corresponding to 8 murine proteins) were measured using the MRM transitions described in Table S2. A) Multiplexed detection of each murine tryptic peptide. Example data is from mock infection and a single MRM transition is shown for each peptide for clarity. * indicates false-positive signal peaks. Protein name and peptide sequence (in parentheses) are indicated for each peak. B) Detection levels (calculated from sum MRM area per peptide) of each murine peptide during the infection timecourse relative to the level observed from mock infection. C) Murine tryptic peptide data plotted as individual points showing mean +/− SEM. Cell count (plotted relative to mock on the same scale) is overlaid. D) Normalisation factor (mock set to 1) for each step of the time course calculated by taking the inverse of the detection level relative to mock from (C). Data shows mean +/− SEM, where the mean was used as the normalisation value for calculating VACV protein abundance. The full protein descriptors are defined in Table S2.
(TIF)

Figure S4 **Kinetics of VACV protein expression during infection.** VACV proteins were detected using the MRM transitions described in Table S3. A) Raw (non-normalised) MRM detection intensities at each stage of infection. MRM trace y-axes are set to the maximum detected value for each protein. B) Raw and normalised (see Supporting Information Figure S3) VACV protein levels, plotted as a percentage of maximum.
(TIF)

Figure S5 **Surface MHC class I K^b^ levels during VACV WR infection.** DC2.4 cells were infected with VACV strain WR and incubated for the indicated times at which point cell surface MHC class I K^b^ molecules were visualised by flow cytometry. Data is plotted as the percentage expression relative to mock-infected cells at time 0. Values are mean of triplicate samples +/− SEM and are representative of one of two independent experiments.
(TIF)

Figure S6 **Epitope stabilisation assay of VACV peptides.** A) RMA-S cells were grown overnight at 26°C to induce maximal empty class I expression and then exogenously labelled with the indicated titrated concentrations of each synthetic VACV peptide for 1 hour and then transferred to 37°C for 2 hours. Stabilised cell surface MHC class I complexes were visualised by flow cytometry. B) Epitope stabilisation across 6.5 hours was carried out following labelling of stabilised empty class I molecules with 1 µM of synthetic peptide, washing cells and incubating at 37°C for the indicated times. Stabilised cell surface MHC class I complexes were visualised by flow cytometry. Estimated half-lives for each peptide-MHC complex are as follows: B8_20–27_, 5 hrs; J3_289–296_, 5 hrs; A47_138–146_, 5.5 hrs; A19_47–55_, 7 hrs; L2_53–61_, 12 hrs; A3_270–277_, 5 hrs; A8_189–196_, 5 hrs; C4_125–132_, 4.5 hrs. All data are mean of triplicate values +/− SEM and are representative of at least two independent experiments.
(TIF)

Table S1 **MRM transitions used to monitor for VACV epitopes.** Target epitope position with each protein is indicated, along with epitope amino acid sequence, Q1 and Q3 m/z, the dwell time that the QTRAP instruments spends on each transition and the optimal collision energy (CE) for each transition.
(DOCX)

Table S2 **MRM transitions used to monitor for murine tryptic peptides.** Target protein and peptide amino acid sequence is indicated, along with the Q1 and Q3 m/z, the dwell time that the QTRAP instruments spends on each transition and the optimal collision energy (CE) for each transition.
(DOCX)

Table S3 **MRM transitions used to monitor for VACV tryptic peptides.** Target protein and peptide amino acid sequence is indicated, along with the Q1 and Q3 m/z, the dwell time that the QTRAP instruments spends on each transition and the optimal collision energy (CE) for each transition.
(DOCX)
