# Supplementary figures and images for: Kinetics of Antigen Expression and Epitope Presentation during Virus Infection
Source: PLoS Pathog. 2013 Jan 31;9(1):e1003129. doi: 10.1371/journal.ppat.1003129 (PMC3561264; doi:10.1371/journal.ppat.1003129)

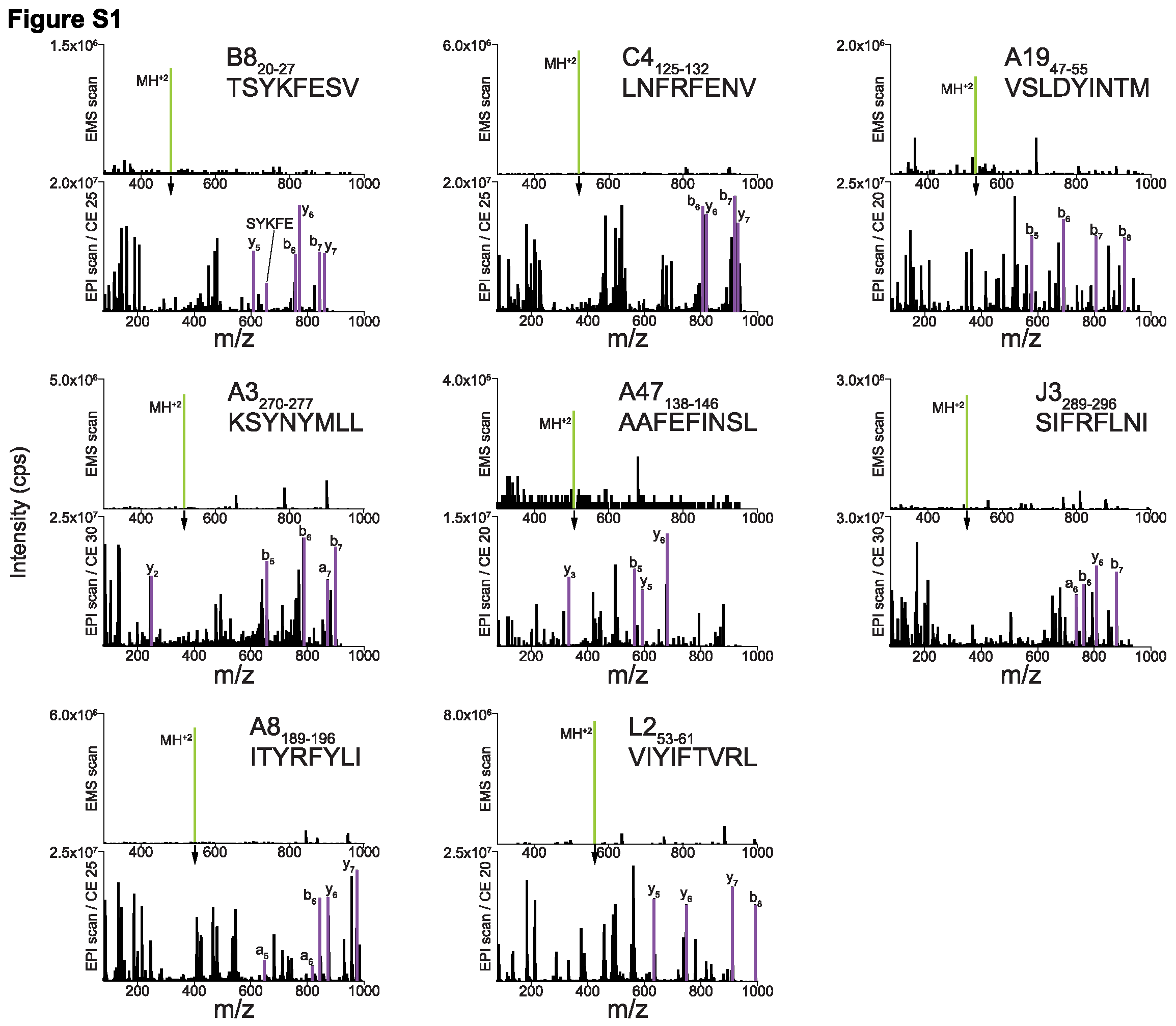

Supplement: Figure S1 — MS and MS/MS analysis of synthetic VACV peptides for MRM design. 200 fmol of each VACV peptide was analysed individually by a QTRAP 5500 operating in enhanced MS (EMS) mode in order to determine the dominant precursor ion (upper panel for each peptide; precursor ion charge is indicated). Subsequently, a full enhanced product ion (EPI) scan (80–1000 m/z range) across a range of collision energy (CE) values was triggered following detection of the dominant precursor (lower panel for each peptide; a single CE is shown for clarity, although different CEs were used for optimal product generation – refer to Table S1). Further refinement of optimal MRM conditions was achieved using a method adapted from Sherwood et al. (Sherwood et al., 2009). At least 4 Q3 product ions (purple lines) were chosen per peptide in order to practically eliminate false-positive signal due to the presence of isobaric peptide species in MHC eluates. (TIF) [file ppat.1003129.s001.tif]

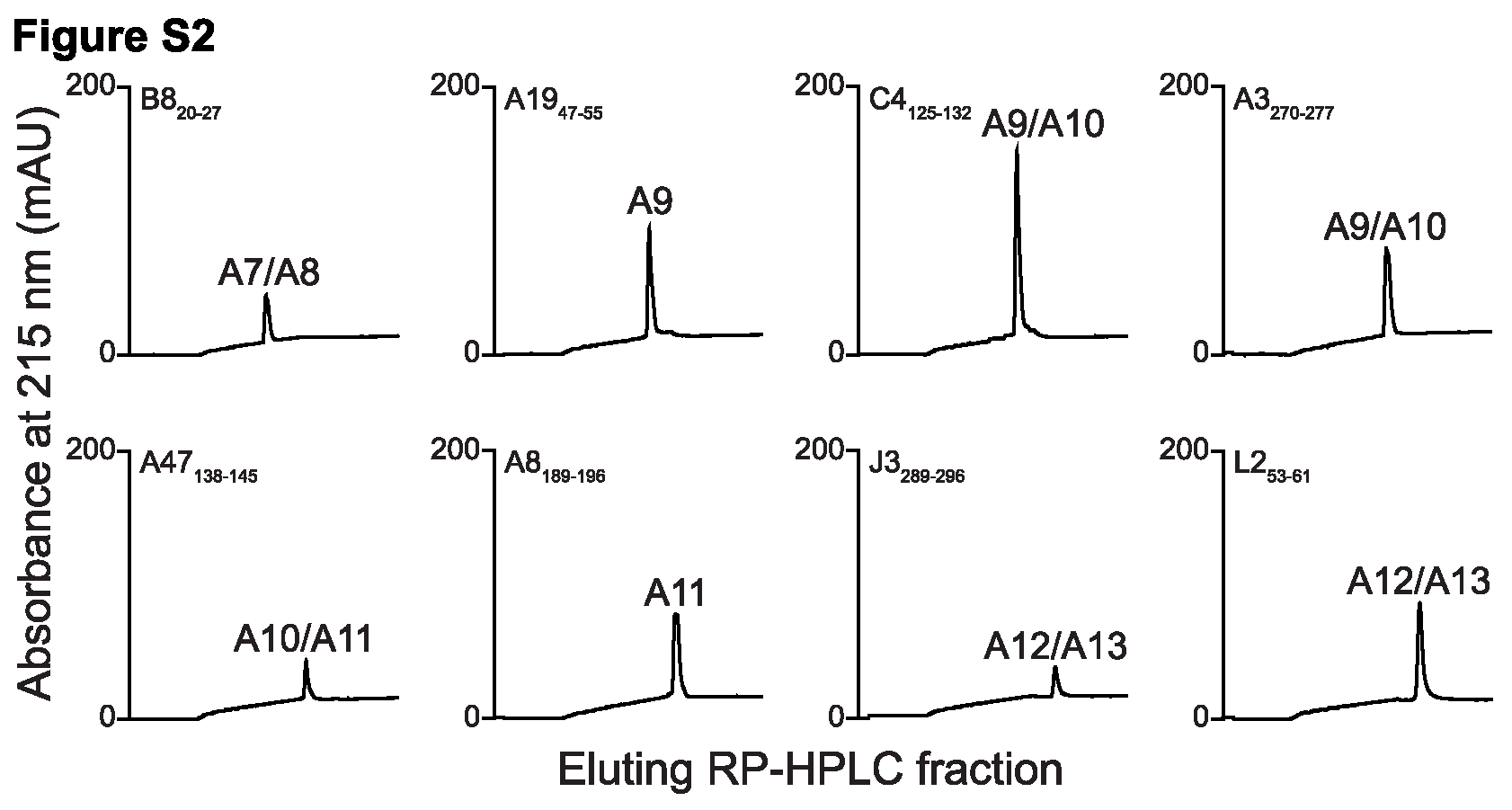

Supplement: Figure S2 — Eluting RP-HPLC fractions for each epitope. 1 nmol of each peptide was spiked individually onto a 4.6 mm internal diameter×50 mm long reversed-phase C18 HPLC column (Chromolith Speed Rod, Merck) using an ÄKTAmicro HPLC system (GE Healthcare) running on a mobile phase buffer A of 0.1% trifluoroacetic acid (TFA) and buffer B of 80% acetonitrile/0.1% TFA and at a flow rate of 1 ml/min. These conditions are identical to those used in the separation of peptides following MHC elution and therefore determines the RP-HPLC fraction (indicated above each chromatographic peak) which contains each VACV epitope. Peptides were read at an absorbance of 215 nm. (TIF) [file ppat.1003129.s002.tif]

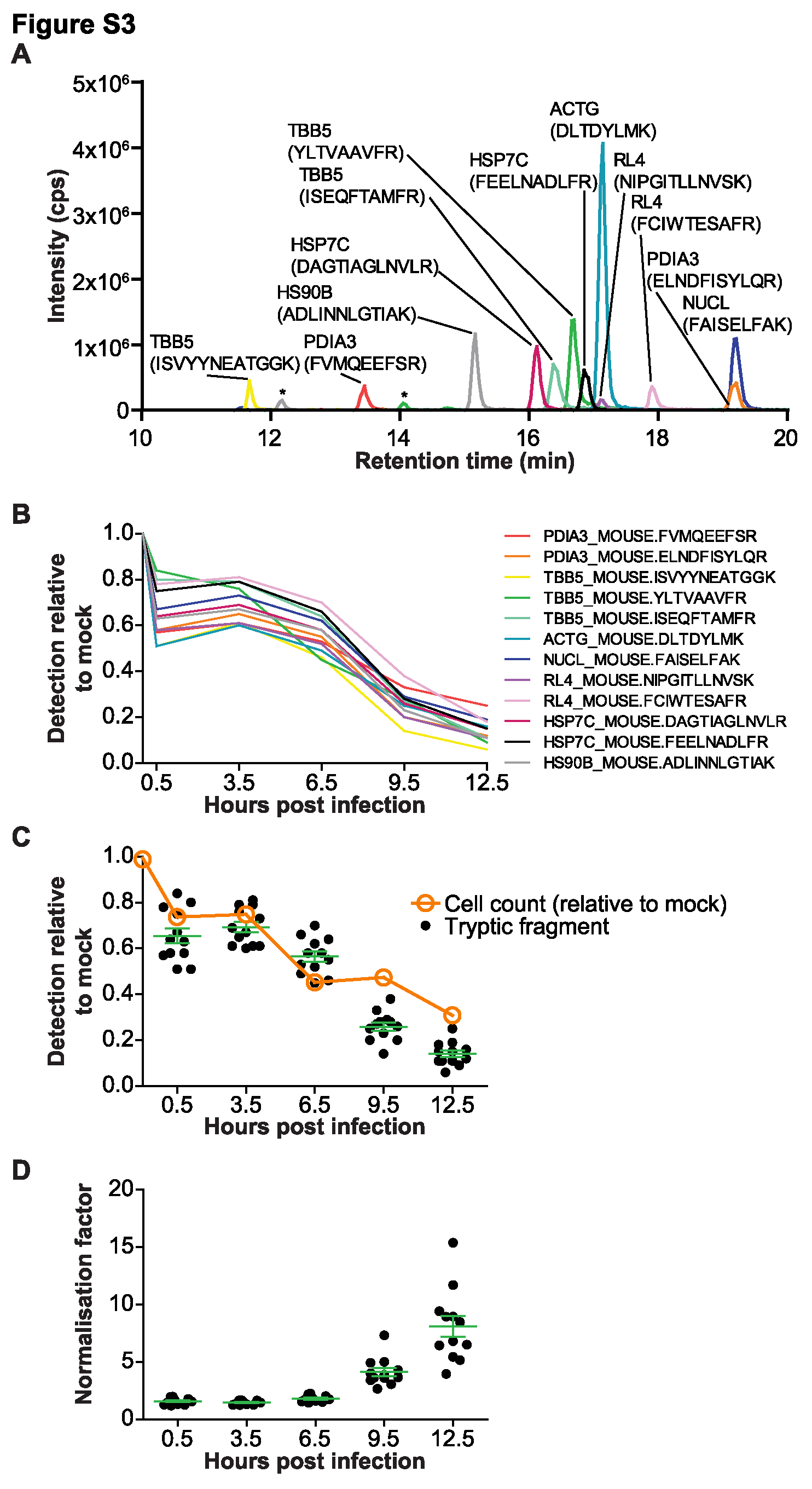

Supplement: Figure S3 — The use of murine protein MRMs for the protein normalisation during VACV infection. In order to accurately measure relative levels of VACV protein expression during infection (see Supporting Information Figure S4) it was necessary to concurrently measure host cellular protein levels as references for normalisation. Following protein lysate reduction and alkylation with iodoacetamide, levels of twelve tryptic peptides (corresponding to 8 murine proteins) were measured using the MRM transitions described in Table S2. A) Multiplexed detection of each murine tryptic peptide. Example data is from mock infection and a single MRM transition is shown for each peptide for clarity. * indicates false-positive signal peaks. Protein name and peptide sequence (in parentheses) are indicated for each peak. B) Detection levels (calculated from sum MRM area per peptide) of each murine peptide during the infection timecourse relative to the level observed from mock infection. C) Murine tryptic peptide data plotted as individual points showing mean +/− SEM. Cell count (plotted relative to mock on the same scale) is overlaid. D) Normalisation factor (mock set to 1) for each step of the time course calculated by taking the inverse of the detection level relative to mock from (C). Data shows mean +/− SEM, where the mean was used as the normalisation value for calculating VACV protein abundance. The full protein descriptors are defined in Table S2. (TIF) [file ppat.1003129.s003.tif]

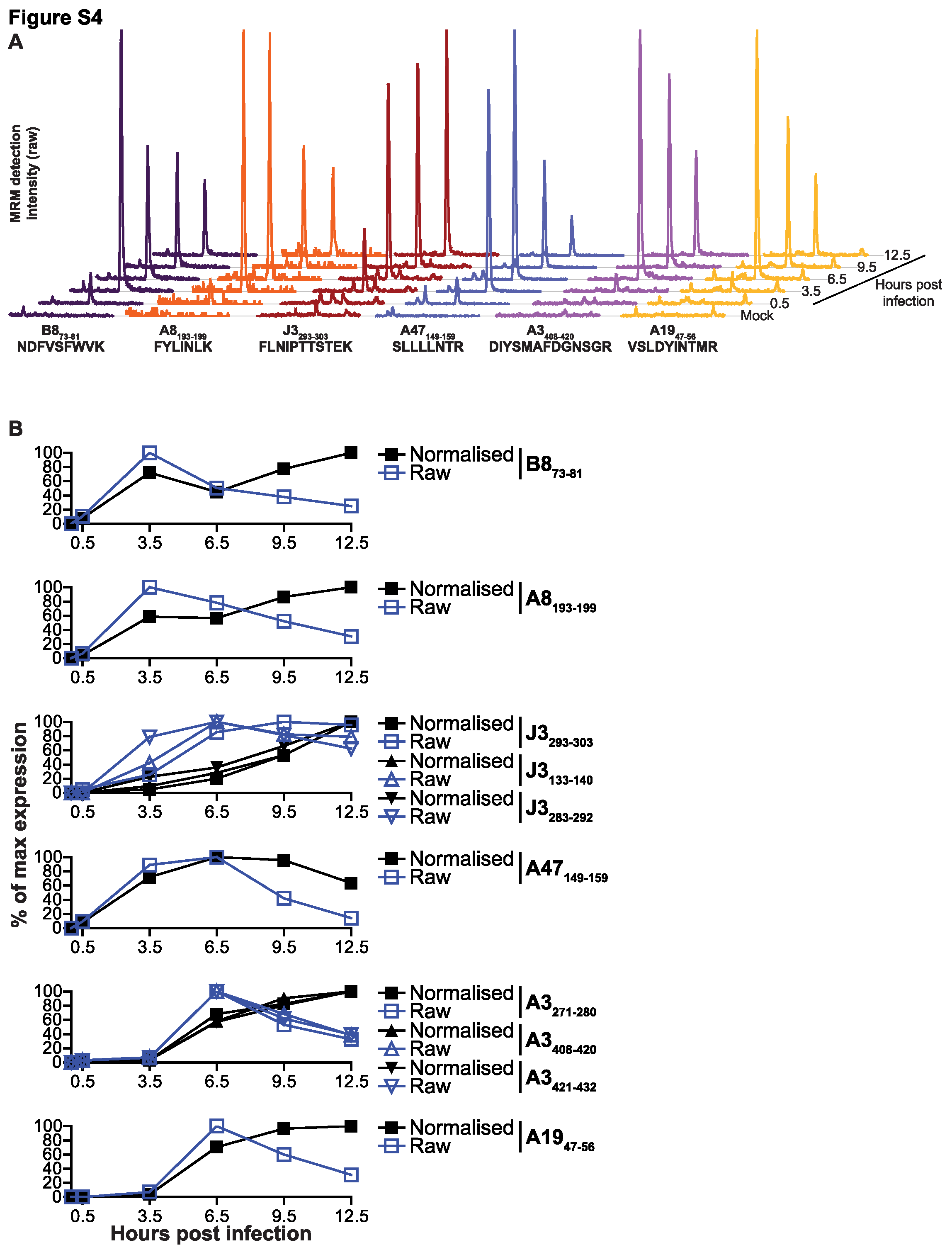

Supplement: Figure S4 — Kinetics of VACV protein expression during infection. VACV proteins were detected using the MRM transitions described in Table S3. A) Raw (non-normalised) MRM detection intensities at each stage of infection. MRM trace y-axes are set to the maximum detected value for each protein. B) Raw and normalised (see Supporting Information Figure S3) VACV protein levels, plotted as a percentage of maximum. (TIF) [file ppat.1003129.s004.tif]

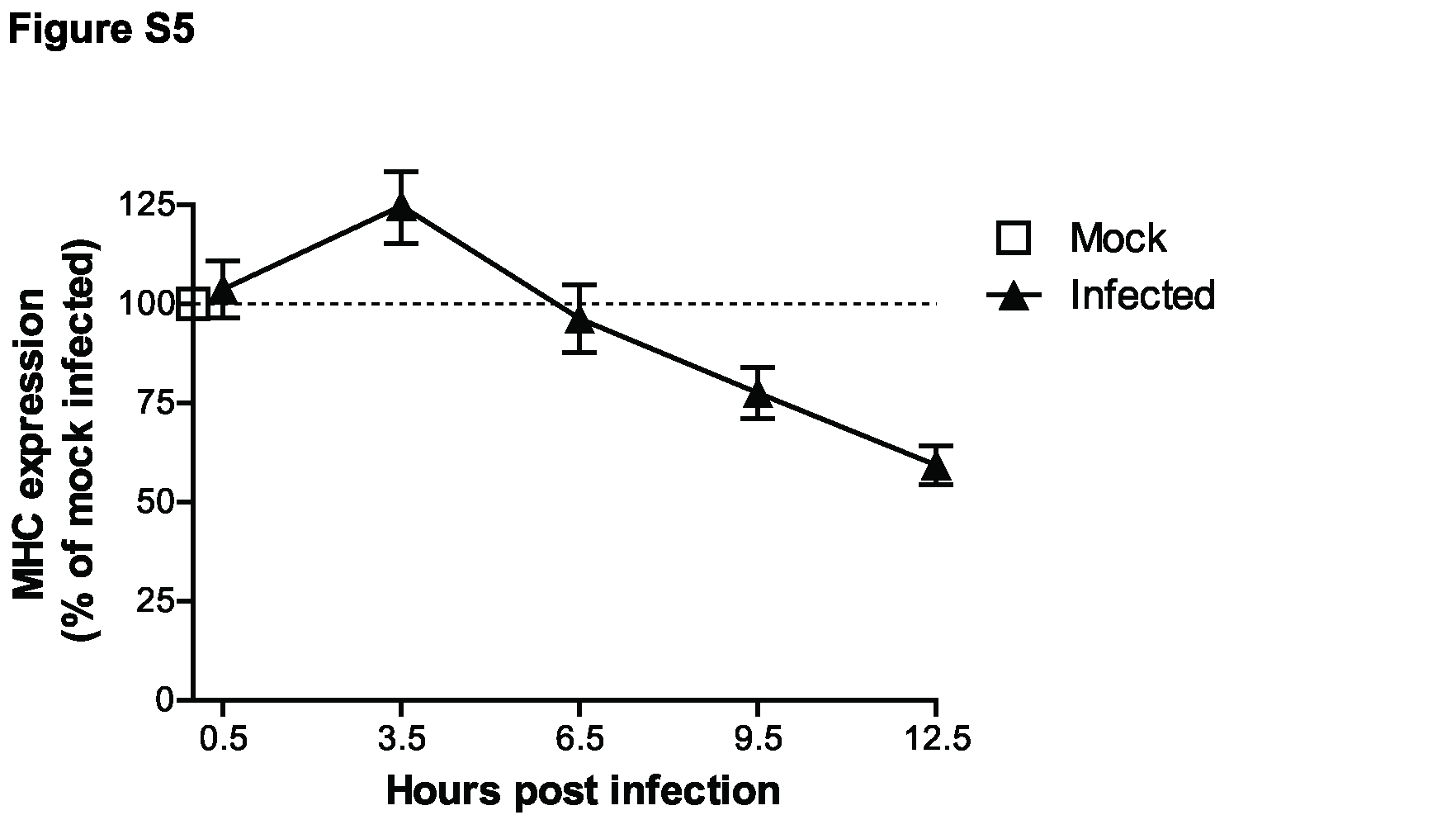

Supplement: Figure S5 — Surface MHC class I Kb levels during VACV WR infection. DC2.4 cells were infected with VACV strain WR and incubated for the indicated times at which point cell surface MHC class I Kb molecules were visualised by flow cytometry. Data is plotted as the percentage expression relative to mock-infected cells at time 0. Values are mean of triplicate samples +/− SEM and are representative of one of two independent experiments. (TIF) [file ppat.1003129.s005.tif]

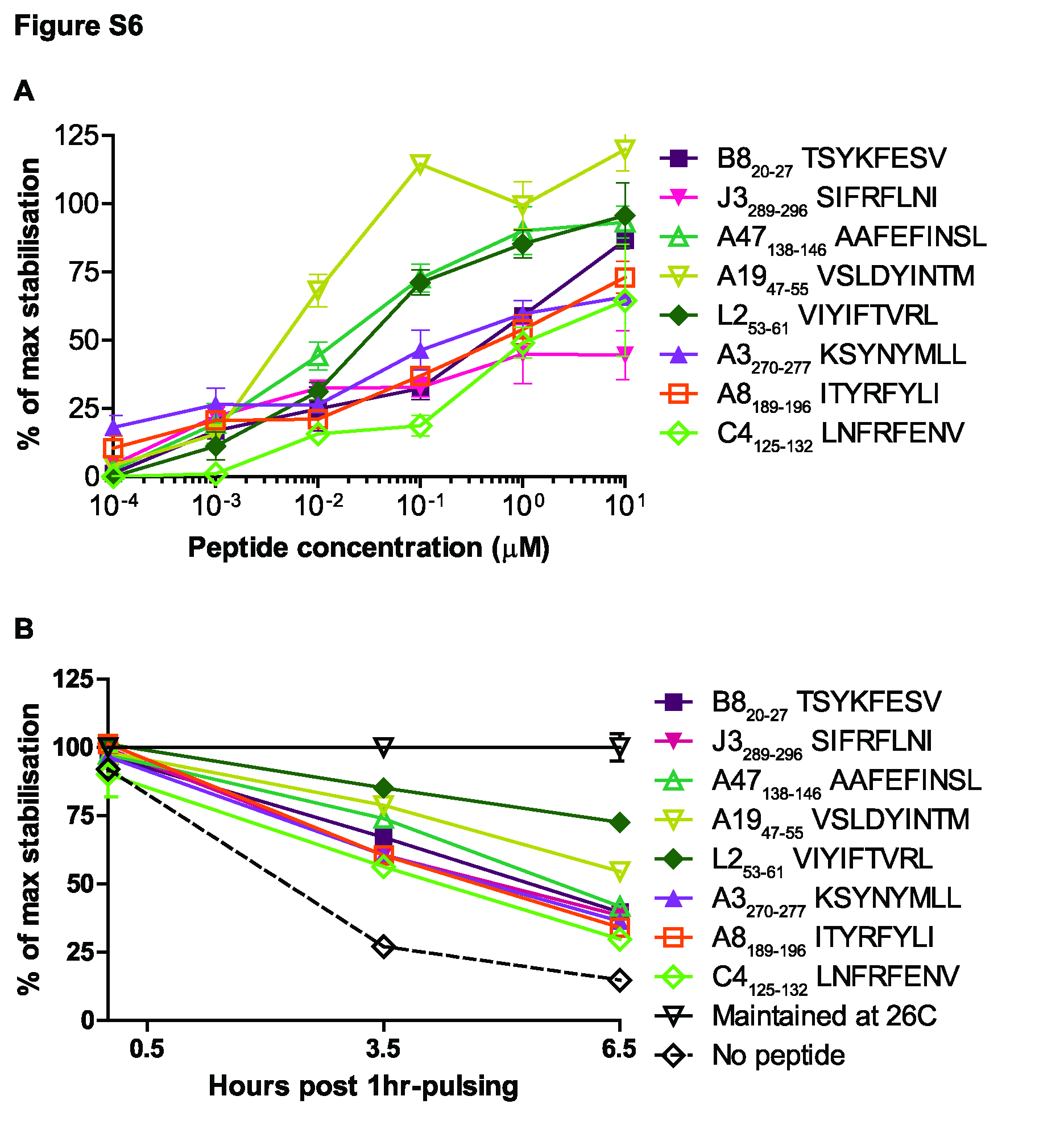

Supplement: Figure S6 — Epitope stabilisation assay of VACV peptides. A) RMA-S cells were grown overnight at 26°C to induce maximal empty class I expression and then exogenously labelled with the indicated titrated concentrations of each synthetic VACV peptide for 1 hour and then transferred to 37°C for 2 hours. Stabilised cell surface MHC class I complexes were visualised by flow cytometry. B) Epitope stabilisation across 6.5 hours was carried out following labelling of stabilised empty class I molecules with 1 µM of synthetic peptide, washing cells and incubating at 37°C for the indicated times. Stabilised cell surface MHC class I complexes were visualised by flow cytometry. Estimated half-lives for each peptide-MHC complex are as follows: B820–27, 5 hrs; J3289–296, 5 hrs; A47138–146, 5.5 hrs; A1947–55, 7 hrs; L253–61, 12 hrs; A3270–277, 5 hrs; A8189–196, 5 hrs; C4125–132, 4.5 hrs. All data are mean of triplicate values +/− SEM and are representative of at least two independent experiments. (TIF) [file ppat.1003129.s006.tif]
