# Supplementary material for: Kinetics of Antigen Expression and Epitope Presentation during Virus Infection
Source: PLoS Pathog. 2013 Jan 31;9(1):e1003129. doi: 10.1371/journal.ppat.1003129 (PMC3561264; doi:10.1371/journal.ppat.1003129)
Supplement: Table S1 — MRM transitions used to monitor for VACV epitopes. Target epitope position with each protein is indicated, along with epitope amino acid sequence, Q1 and Q3 m/z, the dwell time that the QTRAP instruments spends on each transition and the optimal collision energy (CE) for each transition. (DOCX) [file ppat.1003129.s007.docx]

**Supporting Information Table S1 – MRM transitions for VACV epitopes**

| **Epitope** | **Sequence** | **Q1 m/z (charge)** | **Q3 m/z (ion)** | **Dwell time (ms)** | **Optimal CE (Collision energy)** |
| --- | --- | --- | --- | --- | --- |
| B8_20-27_ | TSYKFESV | 480.7 (+2) | 609.3 (y_5_) | 25 | 28 |
|  |  |  | 655.3 (SYKFE) | 25 | 30 |
|  |  |  | 756.4 (b_6_) | 25 | 22 |
|  |  |  | 772.4 (y_6_) | 25 | 23 |
|  |  |  | 843.4 (b_7_) | 25 | 22 |
|  |  |  | 859.4 (y_7_) | 25 | 25 |
| A8_189-196_ | ITYRFYLI | 544.8 (+2) | 653.4 (a_5_) | 25 | 43 |
|  |  |  | 816.4 (a_6_) | 25 | 38 |
|  |  |  | 844.4 (b_6_) | 25 | 25 |
|  |  |  | 874.5 (y_6_) | 25 | 30 |
|  |  |  | 975.5 (y_7_) | 25 | 30 |
| A3_270-277_ | KSYNYMLL | 516.3 (+2) | 245.2 (y_2_) | 25 | 22 |
|  |  |  | 656.3 (b_5_) | 25 | 31 |
|  |  |  | 787.3 (b_6_) | 25 | 23 |
|  |  |  | 872.4 (a_7_) | 25 | 30 |
|  |  |  | 900.4 (b_7_) | 25 | 26 |
| C4_125-132_ | LNFRFENV | 519.8 (+2) | 807.4 (b_6_) | 25 | 24 |
|  |  |  | 811.4 (y_6_) | 25 | 22 |
|  |  |  | 921.5 (b_7_) | 25 | 25 |
|  |  |  | 925.5 (y_7_) | 25 | 31 |
| A47_138-146_ | AAFEFINSL | 506.3 (+2) | 333.2 (y_3_) | 25 | 16 |
|  |  |  | 566.3 (b_5_) | 25 | 18 |
|  |  |  | 593.3 (y_5_) | 25 | 18 |
|  |  |  | 679.3 (b_6_) | 25 | 16 |
| L2_53-61_ | VIYIFTVRL | 562.3 (+2) | 635.4 (y_5_) | 25 | 36 |
|  |  |  | 748.5 (y_6_) | 25 | 33 |
|  |  |  | 911.5 (y_7_) | 25 | 33 |
|  |  |  | 488.5 (y_4_) | 25 | 29 |
| J3_289-296_ | SIFRFLNI | 505.3 (+2) | 736.5 (a_6_) | 25 | 35 |
|  |  |  | 764.5 (b_6_) | 25 | 22 |
|  |  |  | 809.5 (y_6_) | 25 | 22 |
|  |  |  | 878.5 (b_7_) | 25 | 26 |
| A19_47-55_ | VSLDYINTM | 528.3 (+2) | 578.3 (b_5_) | 25 | 16 |
|  |  |  | 691.4 (b_6_) | 25 | 16 |
|  |  |  | 805.4 (b_7_) | 25 | 18 |
|  |  |  | 906.5 (b_8_) | 25 | 18 |
