# Supplementary material for: Kinetics of Antigen Expression and Epitope Presentation during Virus Infection
Source: PLoS Pathog. 2013 Jan 31;9(1):e1003129. doi: 10.1371/journal.ppat.1003129 (PMC3561264; doi:10.1371/journal.ppat.1003129)
Supplement: Table S2 — MRM transitions used to monitor for murine tryptic peptides. Target protein and peptide amino acid sequence is indicated, along with the Q1 and Q3 m/z, the dwell time that the QTRAP instruments spends on each transition and the optimal collision energy (CE) for each transition. (DOCX) [file ppat.1003129.s008.docx]

**Supporting Information Table S2 – Murine tryptic protein MRMs**

| **Protein** | **Tryptic peptide sequence (position in protein)** | **Q1 m/z (charge)** | **Q3 m/z (ion)** | **Dwell time (ms)** | **Optimal CE (collision energy)** |
| --- | --- | --- | --- | --- | --- |
| PDIA3_MOUSE  (Protein disulfide-isomerase A3) | FVMQEEFSR | 586.8 (+2) | 926.4 (y_7_) | 5 | 29.2 |
|  |  |  | 795.4 (y_6_) | 5 | 29.2 |
|  |  |  | 667.3 (y_5_) | 5 | 29.2 |
|  |  |  | 538.3 (y_4_) | 5 | 29.2 |
|  | ELNDFISYLQR | 699.4 (+2) | 1041.5 (y_8_) | 5 | 35.6 |
|  |  |  | 926.5 (y_7_) | 5 | 35.6 |
|  |  |  | 779.4 (y_6_) | 5 | 35.6 |
|  |  |  | 666.4 (y_5_) | 5 | 35.6 |
| TBB5_MOUSE  (Tubulin beta-5 chain) | ISVYYNEATGGK | 651.3 (+2) | 1002.5 (y_9_) | 5 | 32.9 |
|  |  |  | 839.4 (y_8_) | 5 | 32.9 |
|  |  |  | 676.3 (y_7_) | 5 | 32.9 |
|  |  |  | 562.3 (y_6_) | 5 | 32.9 |
|  | YLTVAAVFR | 520.3 (+2) | 763.4 (y_7_) | 5 | 25.4 |
|  |  |  | 662.4 (y_6_) | 5 | 25.4 |
|  |  |  | 563.3 (y_5_) | 5 | 25.4 |
|  |  |  | 492.3 (y_4_) | 5 | 25.4 |
|  | ISEQFTAMFR | 615.3 (+2) | 900.4 (y_7_) | 5 | 30.8 |
|  |  |  | 772.4 (y_6_) | 5 | 30.8 |
|  |  |  | 625.3 (y_5_) | 5 | 30.8 |
|  |  |  | 524.3 (y_4_) | 5 | 30.8 |
| ACTG_MOUSE  (Actin, cytoplasmic 2) | DLTDYLMK | 499.7 (+2) | 770.4 (y_6_) | 5 | 24.2 |
|  |  |  | 669.3 (y_5_) | 5 | 24.2 |
|  |  |  | 554.3 (y_4_) | 5 | 24.2 |
|  |  |  | 391.2 (y_3_) | 5 | 24.2 |
| NUCL_MOUSE  (Nucleolin) | FAISELFAK | 513.3 (+2) | 807.5 (y_7_) | 5 | 25.0 |
|  |  |  | 694.4 (y_6_) | 5 | 25.0 |
|  |  |  | 607.3 (y_5_) | 5 | 25.0 |
|  |  |  | 478.3 (y_4_) | 5 | 25.0 |
| RL4_MOUSE  (60S ribosomal protein L4) | NIPGITLLNVSK | 634.9 (+2) | 887.6 (y_8_) | 5 | 31.9 |
|  |  |  | 774.5 (y_7_) | 5 | 31.9 |
|  |  |  | 673.4 (y_6_) | 5 | 31.9 |
|  |  |  | 560.3 (y_5_) | 5 | 31.9 |
|  | FCIWTESAFR | 658.8 (+2) | 1009.5 (y_8_) | 5 | 33.3 |
|  |  |  | 896.4 (y_7_) | 5 | 33.3 |
|  |  |  | 710.3 (y_6_) | 5 | 33.3 |
|  |  |  | 609.3 (y_5_) | 5 | 33.3 |
| HSP7C_MOUSE  (Heat shock cognate  71 kDa protein) | DAGTIAGLNVLR | 600.3 (+2) | 742.5 (y_7_) | 5 | 30.0 |
|  |  |  | 671.4 (y_6_) | 5 | 30.0 |
|  |  |  | 614.4 (y_5_) | 5 | 30.0 |
|  |  |  | 501.3 (y_4_) | 5 | 30.0 |
|  | FEELNADLFR | 627.3 (+2) | 977.5 (y_8_) | 5 | 31.5 |
|  |  |  | 848.5 (y_7_) | 5 | 31.5 |
|  |  |  | 735.4 (y_6_) | 5 | 31.5 |
|  |  |  | 621.3 (y_5_) | 5 | 31.5 |
| HS90B_MOUSE  (Heat shock protein  HSP 90-beta) | ADLINNLGTIAK | 621.9 (+2) | 943.6 (y_9_) | 5 | 31.2 |
|  |  |  | 830.5 (y_8_) | 5 | 31.2 |
|  |  |  | 716.4 (y_7_) | 5 | 31.2 |
|  |  |  | 602.4 (y_6_) | 5 | 31.2 |
