# Supplementary material for: Kinetics of Antigen Expression and Epitope Presentation during Virus Infection
Source: PLoS Pathog. 2013 Jan 31;9(1):e1003129. doi: 10.1371/journal.ppat.1003129 (PMC3561264; doi:10.1371/journal.ppat.1003129)
Supplement: Table S3 — MRM transitions used to monitor for VACV tryptic peptides. Target protein and peptide amino acid sequence is indicated, along with the Q1 and Q3 m/z, the dwell time that the QTRAP instruments spends on each transition and the optimal collision energy (CE) for each transition. (DOCX) [file ppat.1003129.s009.docx]

**Supporting Information Table S3 – VACV tryptic protein MRMs**

| **Protein** | **Tryptic peptide sequence (position in protein)** | **Q1 m/z (charge)** | **Q3 m/z (ion)** | **Dwell time (ms)** | **Optimal CE (collision energy)** |
| --- | --- | --- | --- | --- | --- |
| A47 | SLLLLNTR (149-159) | 465.3 (+2) | 729.5 (y_6_) | 10 | 25.0 |
|  |  |  | 616.4 (y_5_) | 10 | 25.0 |
|  |  |  | 503.3 (y_4_) | 10 | 25.0 |
|  |  |  | 173.1 (a_2_) | 10 | 25.0 |
| A8 | FYLINLK (193-199) | 455.8 (+2) | 763.5 (y_6_) | 10 | 25.3 |
|  |  |  | 600.4 (y_5_) | 10 | 25.3 |
|  |  |  | 487.3 (y_4_) | 10 | 25.3 |
|  |  |  | 374.2 (y_3_) | 10 | 25.3 |
| B8 | NDFVSFWVK (73-81) | 571.3 (+2) | 912.5 (y_7_) | 10 | 28.3 |
|  |  |  | 765.4 (y_6_) | 10 | 28.3 |
|  |  |  | 666.4 (y_5_) | 10 | 28.3 |
|  |  |  | 377.1 (b_3_) | 10 | 28.3 |
| J3 | FLNIPTTSTEK (293-303) | 625.8 (+2) | 990.5 (y_9_) | 10 | 34.0 |
|  |  |  | 763.4 (y_7_) | 10 | 34.0 |
|  |  |  | 375.2 (b_3_) | 10 | 34.0 |
|  |  |  | 233.2 (a_2_) | 10 | 34.0 |
|  | IILISDVR (133-140) | 464.8 (+2) | 702.4 (y_6_) | 10 | 25.5 |
|  |  |  | 589.3 (y_5_) | 10 | 25.5 |
|  |  |  | 343.2 (y_6_-NH_3_^+2^) | 10 | 25.5 |
|  | VLFLQQSIFR (283-292) | 625.9 (+2) | 891.5 (y_7_) | 10 | 33.5 |
|  |  |  | 778.4 (y_6_) | 10 | 33.5 |
|  |  |  | 519.8 (y_8_^+2^) | 10 | 33.5 |
| A3 | SYNYMLLVNR (271-280) | 636.8 (+2) | 908.5 (y_7_) | 10 | 32.0 |
|  |  |  | 745.4 (y_6_) | 10 | 32.0 |
|  |  |  | 614.4 (y_5_) | 10 | 32.0 |
|  | DIYSMAFDGNSGR (408-420) | 716.8 (+2) | 823.4 (y_8_) | 10 | 36.6 |
|  |  |  | 752.3 (y_7_) | 10 | 36.6 |
|  |  |  | 605.3 (y_6_) | 10 | 36.6 |
|  | VVFAPPNIGYGR (421-432) | 654.4 (+2) | 944.5 (y_9_) | 10 | 35.0 |
|  |  |  | 873.5 (y_8_) | 10 | 35.0 |
|  |  |  | 437.2 (y_8_^+2^) | 10 | 35.0 |
| A19 | VSLDYINTMR (47-56) | 606.3 (+2) | 912.4 (y_7_) | 10 | 32.0 |
|  |  |  | 797.4 (y_6_) | 10 | 32.0 |
|  |  |  | 634.3 (y_5_) | 10 | 32.0 |
|  |  |  | 521.3 (y_4_) | 10 | 32.0 |
